# The Emerging Role of Cyclin-Dependent Kinase Inhibitors in Treating Diet-Induced Obesity: New Opportunities for Breast and Ovarian Cancers?

**DOI:** 10.3390/cancers14112709

**Published:** 2022-05-30

**Authors:** Reyes Benot-Dominguez, Annamaria Cimini, Daniela Barone, Antonio Giordano, Francesca Pentimalli

**Affiliations:** 1 Sbarro Institute for Cancer Research and Molecular Medicine and Center for Biotechnology, College of Science and Technology, Temple University, Philadelphia, PA 19122, USA; tun45879@temple.edu (R.B.-D.); giordano@temple.edu (A.G.); 2 Department of Life, Health and Environmental Sciences, University of L’Aquila, 67100 L’Aquila, Italy; annamaria.cimini@univaq.it; 3 Cell Biology and Biotherapy Unit, Istituto Nazionale Tumori, IRCCS, Fondazione G. Pascale, 80131 Napoli, Italy; d.barone@istitutotumori.na.it; 4 Department of Medical Biotechnologies, University of Siena, 53100 Siena, Italy; 5 Department of Medicine and Surgery, LUM University, 70010 Bari, Italy

**Keywords:** CDK inhibitors (CDKIs), diet-induced obesity (DIO), ovarian cancer, breast cancer, cell-cycle inhibitors, RB1, CDK4/6

## Abstract

**Simple Summary:**

This review aims to provide an outline of the potential use of plant-based foods, nutraceuticals, and derived micronutrients—particularly those typically found in the Mediterranean diet—as anticancer agents, with a focus on their mechanism of action as cyclin-dependent kinase inhibitors (CDKIs) by inactivating the CDK 4/6 pathway and the regulation of the cell-cycle cascade. We discuss the preclinical and pharmacological significance of some already approved CDK blockers as a promising therapeutic approach against breast and ovarian cancers.

**Abstract:**

Overweight and obesity constitute the most impactful lifestyle-dependent risk factors for cancer and have been tightly linked to a higher number of tumor-related deaths nowadays. The excessive accumulation of energy can lead to an imbalance in the level of essential cellular biomolecules that may result in inflammation and cell-cycle dysregulation. Nutritional strategies and phytochemicals are gaining interest in the management of obesity-related cancers, with several ongoing and completed clinical studies that support their effectiveness. At the same time, cyclin-dependent kinases (CDKs) are becoming an important target in breast and ovarian cancer treatment, with various FDA-approved CDK4/6 inhibitors that have recently received more attention for their potential role in diet-induced obesity (DIO). Here we provide an overview of the most recent studies involving nutraceuticals and other dietary strategies affecting cell-cycle pathways, which might impact the management of breast and ovarian cancers, as well as the repurposing of already commercialized chemotherapeutic options to treat DIO.

## 1. Introduction

Cancer constitutes the second leading cause of death globally [1,2,3]. Although the cancer death rate has been steadily declining in the past years owing to improved ability in prevention [3], detection, and also treatment, cancer remains a significant concern. Indeed, cancer is mostly a disease of aging, and the number of people aged 60 years or more is expected to double by 2050 reaching 1.5 billion worldwide [4]. Therefore, huge efforts must be done in cancer prevention. 

Obesity is a deadly preventable disease that has trebled its incidence within the last 45 years, affecting almost 40% of adults and 340 million children and teenagers in 2016 [5]. Excessive body weight and body mass index (BMI) constitute the highest non-inherited risk factors in cancer development. It is estimated that nearly 50% of the most common cancers could be avoided by reducing exposure to high-risk factors and adopting healthy behaviors, including diet and physical activity [6]. Harmful dietary patterns that contribute to fatness, including overeating processed meat, refined sugars, or junk food, have been linked to a major risk of tumor onset (20–30%) and about an 11 to 24% lower chance of cancer survival [7], being even more hazardous in postmenopausal women [8,9]. On the contrary, proper food habits such as those typically adopted in the Mediterranean diet-style patterns—high consumption of legumes, fiber, fruits, and vegetables—are associated with a lower risk of developing malignancies. Additionally, various clinical and preclinical studies point to nutraceuticals and phytonutrients as cheap and available sources of anticancer compounds, which could be used to support cancer care in clinical practice [10].

Obesity is also a well-established risk factor for breast cancer (BC) [11] and ovarian cancer (OC) [12], which are among the most commonly diagnosed tumors in women worldwide, and a leading cause of cancer mortality [13,14,15,16]. Although epidemiologic data have associated obesity with increased risk of cancer development and progression for a variety of tumors long ago, only recently the underlying molecular mechanisms have begun to be characterized [17,18]. The relationship linking fatness and cancer is so strong that certain drugs already FDA approved for DIO are now under study for their potential applications in different cancer types, including breast and ovarian tumors [19,20,21] and vice versa [22].

Cell-cycle deregulation is a hallmark of cancer, and increased expression of cell cycle-related proteins (such as cyclins and cyclin-dependent kinases—CDKs) is a feature of many neoplasms. Recently, potent selective inhibitors of CDK4 and CDK6 have been approved for the treatment of advanced BC and are being tested against other tumor types. Iqbal and colleagues recently showed that obesity-induced deregulation of the cell cycle through retinoblastoma RB1 phosphorylation in hypothalamic neurons that are crucial for energy-balance regulation. Interestingly, they showed that reinstating RB1 function using CDK4/6 inhibitors was an effective treatment against DIO [22,23]. Their seminal work suggests a possible repurposing of pharmacological CDK inhibitors from antitumoral to anti-obesity agents.

Indeed, the increased expression of intrinsic CDKIs such as Cip1/p21 and Kip1/p27 proteins, as well as their enhanced binding to CDKs, have been demonstrated to diminish tumor cell proliferation, invasion and promote apoptosis, and increase patient survival in several tumor types, including BC and OC [24,25,26,27].

Given these premises, a thorough analysis of the shared mechanisms of action in cancer and obesity, with a special deepening in the cell-cycle cascade, is of utmost importance. This review also highlights the last clinical trials (CTs) employing phytochemicals and other dietary interventions in breast and ovarian carcinomas, as well as the most recent and noteworthy Phase II/III trials assessing the use of CDK blockers in these malignancies, which may constitute pivotal treatment strategies for BC and OC.

## 2. Obesity and Cell-Cycle Progression in Cancer

Obesity has become an emergent pandemic involving 1/3 of the population worldwide, a multifaceted disorder characterized by the overabundant accumulation of adipocytes—fat cells—which in turn may aggravate the course of different types of chronic diseases. In fact, more aggressive tumor profiles have been seen in overweight breast cancer patients, where adipocytes can secrete hormones, growth factors, and adipokines and release free fatty acids (FFA). The energy obtained from the β-oxidation of FFA can be used by adipocytes to accelerate tumor cell growth and cancer progression, stimulating the oncogenic signaling and leading to angiogenesis and malignant cell migration [28,29]. Similarly, OC risk has also been correlated with elevated BMI and lipid levels. A significant 16–30% increased predisposition to OC has been identified in obese women, with a major risk assigned to specific histological subtypes—mostly endometrioid and mucinous carcinomas—and postmenopausal patients [30]. This pathological condition is frequently associated with the overexpression of pro-inflammatory factors, cytokines, and adipokines, a process promoted by macrophage infiltration within the adipose tissue and able to exert tumor-promoting effects [31]. Actually, the delivery of pro-inflammatory metabolites in the bloodstream may degenerate into hypothalamus deregulation, cause the loss of energy homeostasis, and bring to the disruption of crucial biological pathways, including those determining cell-cycle regulation (https:/grantome.com/grant/NIH/F30-DK116532-04, accessed on 4 November 2022). As a result, elevated levels of markers of inflammation such as tumor necrosis factor-alpha (TNF-α), interleukins (IL) 1—6-8, plasminogen activator inhibitor 1 (PAI1 or SERPINE1), and C-reactive protein (CRP) are also frequently found in obese patients.

The maintenance of energy and body weight balance are critical processes mainly led by the hypothalamic neurons, which constitute one of the most essential targets for adipokines such as leptin, a product of the obese gene (Ob). Altered levels of leptin and related hormones with a key role in food intake and appetite stimuli—such as ghrelin or insulin—are also generally present among overweight patients [32].

### 2.1. Adipokines' Role in Cell-Cycle Progression

Adipokines are a group of cytokines secreted in the adipose tissue involved in the metabolic signaling in the brain, with an extensively demonstrated function in fostering cancer development. The most likely molecular mediators of inflammation from the adipose tissue itself are the adipokines leptin (discovered in 1994) and adiponectin (described for the first time in 1995)—critical for the maintenance of balanced bodyweight—and pro-inflammatory agents.

#### 2.1.1. Leptin

**Figure 1:**
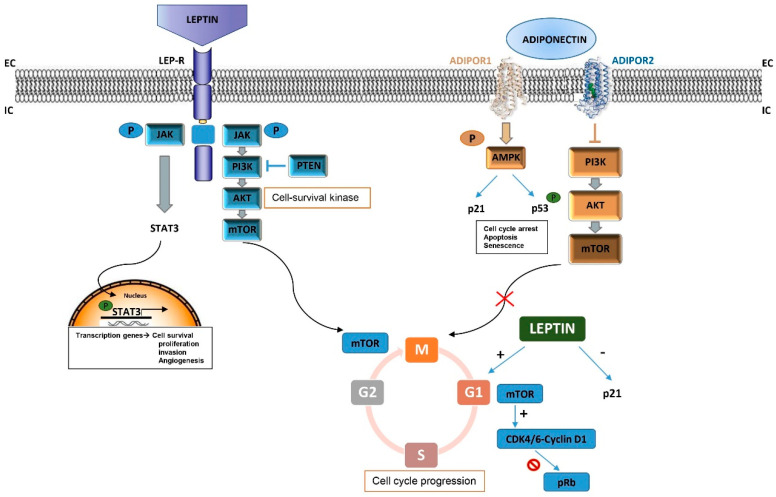
Leptin and Adiponectin’s role on signaling pathways that control cell cycle progression and tumor development.

A wide number of epidemiological studies are focusing on leptin hormone and its receptor (LepR) as good targets for the treatment approach of DIO, and have directly correlated the respective anorexigenic effects with the tumor cascade outgrowth. Leptin has been suggested to strongly take part in cancer onset and proliferation, by activating several growth signaling pathways such as PI3K/Akt, MAPKs (ERK1/2), and JAK/STAT3 [32,33], which drive cell-cycle progression acting on different target genes (Figure 1). These properties joined with the ability of leptin to promote angiogenesis, confer to this adipokine the main role as a growth factor for cancer cells [34]. Indeed, some options presently under study are devoted to inhibiting the leptin cytokine cascade by using multiple approaches, including antibodies [35], peptides [36], and PPAR ligands [37,38]. Some of these leptin antagonists have been seen to efficiently arrest the leptin-induced cell-cycle progression at the S-phase in triple-negative breast cancer (TNBC) [39,40,41].

Ptak et al. examined the relationship between OC development and leptin rates in obese women, identifying a key role of adipokine in stimulating cell cycle-related effectors. The research group connected the tumor proliferation role of leptin with a greater expression of cyclin D—indicative of a poorer prognosis—and cyclin A in an in vitro model; also identifying a downregulation of p21 [33]. These results indicate leptin as a cell-cycle promoter, driving G1/S-phase transition in an OC model. Furthermore, an anti-apoptotic role of leptin was also observed in ovarian carcinogenesis through the blockade of caspase expression, which further stimulates cancer cell proliferation.

The expression levels of leptin constitute an interesting diagnostic tool that can be used to determine cancer risk, grade and type, stage, lymph node involvement, hormone receptors, and prognosis in breast [42,43,44] and ovarian tumors [45]. High leptin levels have been also correlated with lower chemosensitivity, and a common nexus between leptin and several mechanisms that come usually activated in breast tumors—such as VEGF and other pro-angiogenic pathways or estrogen/progesteron receptor signaling—have also been identified [46,47,48,49]. Moreover, LEPR expression has been seen to be able to increase the cancer stem cell state in breast tumors, further promoting cell proliferation, stemness, and poorer survival [50].

Therefore, targeting leptin/LEPR signaling pathways is considered a potential therapeutic strategy for breast and ovarian cancer treatment.

#### 2.1.2. Adiponectin (APN)

In contrast to leptin’s effects on cancer, APN has been demonstrated to exert a protective role in the course of different malignancies, especially BC. APN is gaining interest in the management of obesity, where the extremely low levels of this hormone have been linked to insulin resistance, glucose metabolism, and thermogenesis processes. Interestingly, insulin promotes MCF-7 breast tumor cells proliferation and migration via PI3K activation [51], and APN is able to downregulate the PI3K/Akt/mTOR cascade, resulting in an overall decrease in cancer cell viability, survival, and growth (Figure 1). Focusing on cancer signaling, this adipokine also activates AMPK, which induces cell-cycle arrest, apoptosis, and senescence via p21 activation and p53 phosphorylation (Figure 1). Moreover, APN upregulation also impedes STAT3 signal pathway activation, which in turn is unable to endorse angiogenesis and invasion and incapable of evading anticancer immunity, hence blocking tumor progression. The anti-inflammatory and pro-apoptotic effects of APN also involve the abolition of the NF-kB cascade by hampering NF-kB phosphorylation [52,53].

In an in vitro model of TNBC (MDA-MB-231 cell line), APN enhanced the overexpression of master genes that control cell-cycle progression, such as p53, and apoptosis (BAX, BCL2) [54,55]. This study also showed how the repression of the proto-oncogene MYC prevented cyclin D1 activation, consequently arresting the cell cycle at the G1/S-phase and hampering TNBC expansion. Nonetheless, the antiproliferative effects of APN in mammary cancers seem reliant on ERα expression, as an opposite role was observed in ERα + BC, where APN seems to promote cancer cell growth. On the opposite, low APN levels stimulate MAPK activation, which consequently phosphorylates SP1 and ER and enhances cyclin D1 expression, stimulating BC growth.

Despite the potential ERα-dependent effect of APN, patients with low leptin/APN ratios have shown a statistically longer cancer-specific survival for OC, which may show APN as a good candidate against DIO and derived metabolic diseases, including cancer [56].

### 2.2. Additional Pro-Inflammatory Cytokines

The relationship between chronic inflammation, obesity, and several types of cancer has been extensively investigated, correlating the aggressiveness of tumor disease with higher levels of circulating inflammatory biomarkers such as cytokines [57]. These functional proteins released by immune, stromal, and tumor cells affect cell proliferation via cell-cycle-regulatory proteins. As an example, the transforming growth factor-beta (TGF-β), is a key cytokine that in normal conditions induces tolerance and suppresses inflammation and in the early phases of tumorigenesis acts as a cytostatic tumor suppressive agent acting through p21 and p27 CDKI expression and inducing cancer cell apoptosis (Table 1) [58,59]. During tumor progression, however, TGF-β or its pathway is altered and decoupled from their tumor suppressor activity leading them to promote EMT and favoring a tumor immunosuppressive microenvironment that further enhances tumor invasiveness, as has been seen in HER2- BC [59,60].

Inflammatory cytokines such as IL-6, IL-21, IL-1β, and TNF-α diminish the cytotoxic capacity of immune CD8+ T cells to produce IFN-γ, which plays a main role in angiogenesis and MHC expression—tumor recognition (Table 1). Higher values of these cytokines directly increase IL-17 production, activating angiogenesis and tumor growth [61]. Indeed, risen expression of IL-1, IL-5, IL-6, IL-17, and NFκB were linked to aggressive phenotypes in BC patients and were correlated to a poorer prognosis and lower survival rates [62,63].

Interestingly, IL-6 has been demonstrated to switch on the JAK/STAT3 pathway and enhance EMT, which in turn promotes cancer proliferation, metastasis, and chemoresistance (Table 1). Moreover, IL-6 is the main pro-inflammatory factor responsible for inducing the overexpression of tumor-related RAC1B, known to sustain tumor cell survival and promote escape from oncogene-induced senescence. Finally, increased levels of serum IL-6 have been correlated with poor prognosis, tumor size, and disease status [64]. IL-17 can trigger the production of IL-6, which increases tumor cell migration and invasion, therefore contributing to tumor drug sensitivity and resistance to chemotherapy [65].

IL-8 is also frequently found at high levels in BC, exerting inflammatory and angiogenic actions. In fact, a recently published study has shown that the pro-tumorigenic and metastatic effect of IL-8 passes by the activation of PI3K-Akt/MAPK and EMT signaling pathways leading to tumor cell migration (Table 1) [66]. IL-4 instead upregulates adhesion molecules, inhibits cell proliferation and apoptosis, and mediates signal transduction in breast (MDA-MB-231) and ovarian tumors (SKOV-3), among others [67]. Controversially, it has also been claimed that IL-4 possesses potent antitumor activity against various cancer types, including breast tumors, a reason by which additional research is needed prior to reaching a unique conclusion for this pro-inflammatory factor.

Interleukin-9 (IL-9) is a cytokine with pleiotropic functions that plays an important role in regulating tumor cell growth. IL-9 is increasingly produced by tumor-infiltrating T cells (TILs), as well as tumor cells themselves and a subset of Foxp3 expressing regulatory T cells (Tregs). FoxP3+ Treg cells are known to suppress antitumor immunity, suggesting that IL-9 derived from these cells might control immune responses [68].

IL-10 is an immunosuppressive cytokine that can inhibit the ability of dendritic cells and macrophages to activate CD4 + T cells. IL-10 is frequently present at sites of chronic inflammation, promoting immunosuppression of humoral responses through the induction of isotype switching to IgG4. In a recent study, authors found a significant expression of IL-10 in tumor-infiltrating B-cells of TNBC patients, driving isotype switch to the IgG4 isotype in an IL-10 dependent manner [69].

These observations suggest that IL-10 may play a role in directing antitumor immune escape. Moreover, both IgG4 and tumor IL-10 are associated with shorter recurrence-free survival (RFS) and overall survival (OS). In BC, IL-10 expression positively correlates with locally advanced disease and higher tumor grade and has been proposed as a good prognostic indicator of disease-free survival (DFS) [70]. In melanoma, IL-10 expression by tumor cells is associated with melanoma progression [71], while overexpression of serous IL-10 leads to an adverse survival in most cancer types [72].

Pro-inflammatory stimuli have been seen to be able to raise pro-angiogenic factors in TNBC cells that physically interact with mesenchymal stem cells—MSCs—and stromal cells, accelerating the metastatic phenotype [73]. Additionally, the Notch pathway, probably via CXCL8 cytokine release, has been demonstrated to promote the cell-to-cell interaction, affecting proliferation, differentiation, and death of cells—fostering TNBC spreading and invasion [74]. In the same way, TNF-α has been seen to exert a tumor-promoting role in BC progression and induce metastasis, fostering tumor escape from immune system control (Table 1) [75]. Nonetheless, some studies have recently addressed a controversial role of TNF-α, showing pro-apoptotic and anticarcinogenic functions towards different tumor types, which could also be dependent on TME or specific conditions such as the TNFR that controls the pathway or the ER/PR molecular BC type [76,77].

**Table 1:** Functional roles of pro-inflammatory cytokines in tumor progression and immune response.

Cytokine	Cytokine Family	Activity	References
CXCL8	Chemokines	Cell-to-cell interaction, tumor proliferation, and differentiation Enhances TNBC spreading and invasion	[73]
CXCL14	Chemokines	CXCL14 overexpression is associated with high cancer invasiveness in BC patients	[78,79]
IFNs-1 (IFN-α)	Interferons	Apoptosis induction and repression of malignant tumor progression (via STAT-3)	[80]
IL-2 IL-12	Interleukins	Anticancer activity Tumor size reduction Enhance anticancer immune response by cytotoxic immune cells activation	[58]
IL-4	Interleukins	Exerts antitumor and immunosuppressive action Supports tumor cell spread, migration, and clonogenicity Reduce IFN-γ and TNF-α expression during the inflammatory response	[67]
IL-6	Interleukins	Pro-tumorigenic and anti-apoptotic effects EMT-inducer ROS and RNS release Potential target for NSCLC	[81,82]
IL-8	Interleukins	Promotes inflammation, EMT signaling, and angiogenesis via PI3K-Akt Bad prognostic factor in BC	[66]
IL-9	Interleukins	Enhances tumor progression and causes metastases in BC patients avoiding antitumor immunity	[68,83]
IL-10	Interleukins	Induces immunosuppression and tumor immune evasion Correlates with higher tumor grade and lower survival rates	[71,72]
IL-21, IL-1β, and TNF-α	Interleukins	Reduce IFN-γ production by CD8 + T cells Elevate IL-17 secretion Activate angiogenesis Promote tumor growth	[61,65]
IL-11	Interleukins	Promotes growth in BC and gastric most invasive cancer types	[84]
IL-17	Interleukins	Enhances tumor cell migration and invasion Decreases chemosensitivity and promotes chemoresistance	[85]
TGF-β	Transforming Growth Factors	Affects cell proliferation (also acts on p21 and p27) Often deregulated in cancers where it promotes EMT, immune escape, and angiogenesis, which in turn lead to cancer invasion and metastases and induce anti-apoptotic pathways	[59,60]
TNF-α	Adipokine/TNFR	Induces EMT signaling Cell proliferation and pro-angiogenic role Contributes to the metastasis of BC cells and increases resistance to chemotherapy	[86,87]

## 3. A Common Strategy in Cancer and DIO: Targeting Cell-Cycle Progression/CDKs

Several anti-obesity drugs are being tested for their potential interest as antitumoral agents, including lipid-lowering agents [88]. For instance, the antihyperlipidemic agent orlistat has extensively proven to induce S-phase cell-cycle arrest and apoptosis in BC [89]; whereas recently Harborg et al. carried out a cohort study that explored the link between the use of statins and the risk of developing BC, confirming an indirect relationship between them in postmenopausal early BC patients [90]. Similarly, a noticeable 19% decrease in the OC demises was also noticed in a parallel study comparing mortality among statin users versus patients who never took statins before, with a major benefit assigned to Simvastatin [91]. Furthermore, statin therapy not only did not entail a comparable toxic profile versus chemotherapy, but evidence also supports the ability of these antilipidemic drugs to promote apoptosis in malignant cells, reducing cancer progression and invasiveness [92].

Iqbal et al. (2020) have recently pointed out the importance of approaching the obese state as a neuronal disorder, where RB1 acquires a crucial role as a cell-cycle inhibitor that can be switched off by CDKs [23]. As one of the primordial purposes of oncosuppressors is to avoid cell-cycle progression by directly altering cyclin-dependent kinases expression, and CDK proteins are important players in cell-cycle modulation cascades, novel CDK inhibitors-based strategies have been proposed not only for the management of cancer but also for DIO and vice versa. The activity of these serine/threonine protein kinases is highly dependent on the activation of phase-specific cyclins, and the employment of CDKIs has emerged as an innovative strategy in tumor treatment [25,93,94]. In the same way, multiple plant-derived biomolecules and by-products have shown CDK inhibitory functions raising interest as antitumoral agents [95,96,97].

In the following sections, some of the most relevant clinical studies involving dietary approaches and CDKIs in breast and ovarian carcinomas will be addressed.

### 3.1. Food-Based Approaches in Cancer Therapy

Many anti-DIO strategies based on food intake time restrictions are being tested in vivo to better understand how specific nutritional deprivations affect different types of malignancies. These include several types of periodic fasting and intermittent food supply diets such as time-restricted feeding, short-restricted fasting, short-term starvation, alternate-day fasting, or fasting-mimicking diet (FMD). These fasting and dietary limitations are showing encouraging results in the management of obesity, notably impairing chronic disease burden and cancer onset [98,99].

Moreover, these strategies also represent a good and safe alternative that minimally affects non-tumoral cells, while selectively altering the survival chances of neoplastic cells, mainly by decreasing insulin and related factors, glucose, leptin, and cytokines [100].

The ratio and specific type of macronutrients assumed can importantly change the course of the disease. Dietary patterns with a high content of animal-based proteins were correlated with a major risk of cancer demises compared with feeding habits mainly entailing vegetable-derived proteins [101]. Following this thought, plant-based nutraceuticals are bringing attention as antitumor strategies, and micronutrients and phytochemicals of particular interest are undergoing clinical and preclinical trials in the cancer field. Table 2 highlights some of the most relevant completed and ongoing studies targeting breast and ovarian tumors through different dietary approaches.

To exemplify, more than 50 CTs have investigated the beneficial properties of broccoli-derived molecules (mainly sulforaphane and glucoraphanin) in cancer disease remission [102,103]. Nevertheless, only a few of them have brought into focus the molecular mechanisms involving the downregulation of cell cycle-related proteins such as cyclins and CDKs [104], or the induction of CDKIs and correlated pathways involving signaling cascades such as the mammalian target of rapamycin (mTOR) [105] or STAT 3 (Table 2, [106]).

**Table 2:** Selected clinical trials with nutritional/dietary approaches on breast and ovarian cancer therapy.

Dietary Intervention	Outcomes	N	Therapeutic Intervention	Cancer Type	Phase	References
Structured exercise training plus a Mediterranean diet	Positive results observed in BRCA1/2 mutation carriers regarding BMI, eating habits, physical fitness, and stress levels	69	NO	Breast and ovarian cancer	N/A	[107,108,109]
Usual care plus ketogenic diet (experimental group) or dietary recommendations (control)	Reduced fasting levels of glucose and insulin and increased fasting β-hydroxybutyrate in the ketogenic group Decrease of cancer growth-related factors: CA-125, IGF-1, and IGFBP-1	57	NO	Ovarian and endometrial cancer	N/A	[110]
Tocotrienol (Vit. E)	Improved prognosis and PFS resulted from co-treatment of bevacizumab and tocotrienol in chemotherapy refractory ovarian cancer	60	Bevacizumab	Ovarian cancer	Phase II	[111]
Ascorbic acid (Vit. C) Mixed natural carotenoids with vitamin A vitamin E	Intravenous Vit. C enhanced chemosensitivity and reduced toxicity of chemotherapy	27	Standard chemotherapy (carboplatin and paclitaxel)	Ovarian cancer	Phase II	[112]
Supplementation with sietary nthocyanins	Dietary anthocyanins diminished the inflammatory response and skin toxicity in BC patients undergoing radiotherapy	300	Radiotherapy	Breast cancer	Completed	[113]
Short-term fasting (STF)	STF improves fatigue, side effects, and QOL. Extended randomized CTs presently undergoing to extend the findings to a large-scale study (150 participants). The aim is to investigate the effectiveness of fasting strategies vs. plant-based and healthy diets (low protein, low carbohydrates/sugar)	50	Chemotherapy	Breast and ovarian cancer	Phase I	[114,115]
Fasting-mimicking diet (FMD)	FMD improved clinical response to neoadjuvant chemotherapy, QoL, and illness perception (lower fatigue, nausea, and insomnia; and better emotional, physical, cognitive, and social functioning scores) compared to a regular diet	131	Neoadjuvant chemotherapy	Her2- breast cancer	Phase III	[116,117]
SFX-01 (Sulforaphane)	SFX-01 diminished mammosphere formation efficiency in ER+ primary and metastatic tumor samples by blocking STAT3 activation, both alone and combined with conventional anti-estrogen chemotherapy	68	Fulvestrant Tamoxifen	Breast cancer	Phase II	[106]
Polyphenol-rich dietary supplement (commercial lemon, orange, pomegranate, olive, grape, cocoa, curcuma, and broccoli extracts)	Patients consumed simple phenolics (hydroxytyrosol) and polyphenols (procyanidins, hesperidin, eriocitrin, curcumin, resveratrol, punicalagin, and ellagic acid) enriched diet Cocoa extract also contains the methylxanthines theobromine and caffeine	40	NO	Breast cancer	N/A	[118,119]
Curcumin	i.v. Curcumin plus chemotherapy exerted significantly higher ORR and fewer fatigue symptoms vs. paclitaxel + placebo	150	Paclitaxel	Breast cancer	Phase II	[120]

BC: breast cancer, BMI: body mass index, CA-125: cancer antigen 125, ER: estrogen receptor, IGF-1: insulin-like growth factor 1, IGFBP-1: insulin-like growth factor-binding protein 1, i.v.: intravenously, QoL: quality of life, ORR: overall response rate, PFS: progression-free survival.

Indole-3-carbinol (I3C) represents an additional natural anticancer agent belonging to the same broccoli vegetable family (*Brassicaceae*). It was found to block G1/S cell-cycle progression in breast and endometrial cancers, including MCF-7, BT20, and MDA-MB-231 cell lines [121]. The effective reduction of cyclins D1, E, CDK-2, -4, and -6 and the increase of p21, p27, and p15 expression were also validated in response to I3C treatment [97,122].

Roscovitine constitutes another biological molecule employed in anti-DIO therapy with a key role in cell-cycle modulation. Specifically, its activity results in an accumulation of cells in the G2 phase on (ER-α)+ MCF-7 breast cancer cells, preventing them from entering the next cell cycle [123].

Concomitantly to the inhibition of cell-cycle progression, roscovitine—later commercialized as Seliciclib^®^, a first-generation CDKI—showed a remarkable ability to induce apoptosis via a p53-dependent pathway [124]. Additionally, fangchinoline, an alkaloid isolated from the *Menispermaceae* plant family, has been seen to impede G1/S cell cycle transition in MDA-MB-231 and MCF-7 breast cancer cells. The cell cycle blocking effects of fangchinoline alkaloid were further confirmed by a drop in the levels of cyclins D1, D3, and E; CDK-2, -4, and -6, as well as an increased expression of CDKIs p21 and p27 tumor suppressor proteins [125].

The alkaloid Berberine has proved to have cytotoxic and antiproliferative actions in BC [126] and OC cells [127,128], by targeting the Akt downstream pathway, whereas the flavonoid quercetin (*Quercus* sp.) was able to stop the cell cycle at G1/S and G2/M checkpoints. Downregulation of Quercetin-3-methyl ether significantly prompted cell-cycle arrest at the G2-M phase in MDA-MB-231 and MCF-7 human BC cells, decreasing cell proliferation, invasion, and migration and inducing apoptosis [129,130]. A fall in CDK-2, -6, -7, and cyclins A, D1, and E were also confirmed [131].

Curcumin is widely known to promote cell-cycle arrest at G1/S and G2/M phases and to stimulate the expression of tumor suppressor proteins p53, and the p21 and p27 endogenous CDKIs [132,133]. Preclinical studies indicated a beneficial effect of this *Curcuma longa*-derived polyphenol in reducing severe skin side effects of radiotherapy in BC patients [134]. Furthermore, a synergistic apoptotic action via PARP and p53 activation was seen in combined therapies of curcumin and citral extract, as well as an activation of the oxidative stress signaling via ROS production [135].

Recent clinical data show how multiple dietary changes involving calorie intake reduction or FMDs are able to counteract growth-promoting factors—such as glucose, IGF-1, or insulin—in cancer [136]. Additionally, different kinds of natural compounds have exerted good antimitotic and cell-cycle progression inhibition properties. This anticancer activity could be exploited to include food interventions as co-adjuvants in chemo-, radio- and tumor immunotherapy, as they all have shown anticancer immunity-stimulating functions [137].

### 3.2. Cyclin-Dependent Kinase Inhibitors as Anticancer Drugs

Flavopiridol was the first and most extensively studied CDK inhibitor entering human clinical trials to treat various cancer types, including breast, lung, and bladder [138,139]. This non-selective CDK inhibitor alkaloid initially proved to induce cell-cycle arrest in G0/G1 and an S-phase delay, showing a high specificity against the CDK1/cyclin B complex in BC [140]. Even if the efficacy of flavopiridol in vivo has not been demonstrated to be sufficient to enable it to enter Phase III trials [139] since the FDA approval of this CDKi as an orphan drug for acute myeloid leukemia in 2015, a larger set of molecules have entered clinical testing to evaluate their feasibility in the cancer treatment approach. Indeed, flavopiridol has opened a new window of opportunity for next-generation CDKIs, which means a higher drug specificity by the abolition of cyclin/CDK binding, which consequently impedes the protein complex-associated kinase activity and the subsequent cell-cycle progression. Among these new CDKIs, selective inhibitors of CDK4/6 are particularly gaining the major focus of interest [141], whose activation is mainly dependent on cyclin D-type linkage.

Additional second-generation CDKIs include dinaciclib, a potent inhibitor that targets CDK1, CDK2, CDK5, and CDK9. Despite contrasting results arising, a Phase III randomized study revealed enthralling results in refractory leukemia patients in terms of efficacy, safety, and progression-free survival (PFS) [142]. Moreover, in vitro tests guaranteed the major ability of dinaciclib to suppress Rb phosphorylation versus flavopiridol, subsequently validating a notable cell-cycle arrest in a huge number of malignant cell-based assays [143]. CDK7 inhibitors are also emerging as anticancer therapeutic drugs by targeting diverse pathways, chiefly involving cell-cycle regulators such as CDK-activating kinase, that finally hinder the initiation of oncogenic transcription [93,144]. To date, four different CDK7i are under Phase II studies with encouraging outcomes in breast [145] and ovarian cancers [146,147].

CDK-4 has been identified as a potential blocking target in diet-related anti-obesity treatment, as it promotes adipogenicity [148]. The previously mentioned work of Iqbal and co-workers also described how lipid-enriched diets can induce pRb phosphorylation in the hypothalamus, which consequently inactivates the protein and promotes obesity in vivo. In fact, experiments carried out in mice treated with a first-generation CDKI—abemaciclib—have reported promising results in fat mass reduction and weight loss, and future assays are aimed to deduct which are the molecular mechanisms that may link the abrogation of CDK4/6 and the unphosphorylated form of pRb with the blockade of DIO in neurons [22]. For this purpose, current studies are pointing to anticancer CDK4/6 inhibitors as potential players in the prevention and therapy of DIO via pRb function stimulation [23].

Interestingly, three different CDKIs 4/6 have recently been FDA approved for the treatment of lifelong aggressive and refractory HR+, Her2- BC therapy (palbociclib, PD0332991; ribociclib, LEE011; abemaciclib, LY835219) [149,150,151]. Several biomolecular pathways induced during the obese condition have been demonstrated to be in common with cancer mechanisms of tumor evasion, prompting the study of CDK inhibitors for the management of obesity disease [22]. With this scope, a common strategy linking FDA-authorized CDKIs, nutraceuticals, and dietary approaches could become a feasible tactic to handle overweight-related problems that may potentially favor cancer development.

Palbociclib (PD-0332991) was the first CDKI 4/6 demonstrating a substantial efficacy against breast cancer cells in combination with endocrine therapy in ER+ tumor models in vitro [152]. From 2009 onwards, palbociclib has undergone successful studies in concomitance with hormonal therapies—HT—that led to its FDA approval in 2015 [153], including selective estrogen receptor degraders (SERDs) [154], aromatase inhibitors—AIs—[155], and fulvestrant [156]. Some CTs also show the activity of this CDK4/6 blocker as a single agent both in ovarian [157] and in metastatic breast cancers [158], illustrating a good drug side-effect profile.

Konecny and co-workers (2011) put the basis for the first clinical studies involving palbociclib in ovarian malignancies, corroborating the effectiveness of this second-generation CDKI over a screen of 40 different human tumor cells of several OC subtypes (serous, clear cell, endometrioid, and mucinous) [159]. Cytotoxicity was dose-dependent but showed some variability from one cell line to another; moreover, a direct correlation between p16 hypoexpression, high RB1 levels, and a significant response to palbociclib treatment was verified, both in vitro and in a clinical cohort of 263 OC patients. Inhibition of RB1 phosphorylation and promotion of G1 cell-cycle arrest and apoptosis further supported the promising use of palbociclib in OC, also in later clinical studies [160].

Another noteworthy observation was carried out in TNBC models, a highly aggressive BC subtype characterized by the lack of expression of targetable receptors and a rapid tendency to metastasize to lungs, brain, and bones. Liu and colleagues (2017) performed preclinical studies that enabled them to hypothesize the potential benefit of CDK4/6 inhibition against TNBC invasiveness, where a poor clinical effect had been previously observed. The authors correlated the stunting of tumor growth with the antagonizing role of palbociclib in DUB3-driven CDK4/6 activation, consequently preventing EMT and metastases [161].

Abemaciclib exhibited the highest potency and best delivery efficiency among the three next-generation CDKIs, also showing effects on other kinases such as CDK9 and PIM1 [162,163]. Patnaik et al. (2016) performed preclinical studies in OC human xenografts and patients undergoing abemaciclib therapy [164]. Promising results showed a good safety profile and clinical significance for this CDK 4/6 inhibitor, and a favorable and extended CA-125 response to treatment in advanced OC models. Novel studies concerning abemaciclib monotherapy have also been conducted in HR+/Her2- MBC patients who become refractory to endocrine therapy (Table 3). Among these, the MONARCH-1 trial showed an overall response that accounted for 19.7% of total enrolled patients, whereas clinical benefit exceeded the 42% [165], additionally confirming the antitumor activity and manageable toxicity profile of abemaciclib administered alone [166].

In 2018, the MONALEESA-3 clinical trial outcomes prompted the FDA approval of ribociclib plus fulvestrant in postmenopausal HR+, Her2- advanced BC patients [182]. Following studies of Iyengar and co-workers evaluated the effectiveness of selective CDK4/6 inhibitor ribociclib in different models of high-grade serous ovarian cancer both in vitro and in vivo and identified a pivotal and very selective dose-response activity against cancer cells viability [186]. Cytotoxicity was even more evident upon ribociclib plus cisplatin association, showing a pronounced synergism in co-treatment therapies. The wide ability of CDK4/6 inhibitors to impede cell-cycle progression through the G0/G1 phase was confirmed, whereas the accumulation of cells at the G2/M phase suggested a potential role of ribociclib at this checkpoint as well. Interestingly, the addition of ribociclib also prevented cisplatin chemotherapy-surviving cells to progress over the G2/M cell-cycle phase. Combinations of ribociclib and letrozole also revealed promising results in early HR+ mammary tumors, identifying CDKIs as valuable alternatives to reduce relapses and side effects derived from ET [184,187].

So as the main mechanism underlying G1-targeted CDK4/6 inhibitors go through avoiding RB1 tumor suppressor phosphorylation and its subsequent inactivation, the effects of some agents such as palbociclib require the presence of a functional pRb protein to work properly [188]. Cancers presenting deletions at the pRb protein level represent a treatment challenge, as the lack of a functional target makes these tumors resistant to CDKIs 4/6, making single-agent therapy ineffective [189].

For this reason, CDK 4/6 inhibitors are presently experiencing multiple large, randomized clinical trials to test the prospective combined approaches with anti-estrogen and hormonal therapies [153,171,190,191] (see Table 3). Actually, therapies targeting ER such as tamoxifen, aromatase inhibitors, or fulvestrant that also affect cyclin D1 expression and promote G1 phase cell accumulation may potentiate the blocking function of a CDK4/6 inhibitor in cell-cycle progression [153,155,173]. As a matter of fact, cyclin D1 has strongly exhibited a main role in the development of Her2-driven breast tumors [192].

Intriguing early stage trials are also investigating possible CDKIs and PI3K inhibitors combinations in TNBC. Both palbociclib and ribociclib were administered together with taselisib/alpelisib—respectively—showing a greater synergistic response in terms of cell-cycle arrest and apoptosis compared to single-agent use [193,194].

There are several ongoing and completed Phase II/III studies testing dual CDK4/6 inhibitors against breast and ovarian carcinomas (Table 3), further verifying the antitumor effectiveness of this group of drugs, some of which already manifesting superiority over ET in monotherapy. Additional trials combining palbociclib with other agents (e.g., capecitabine) did not exert comparable results in terms of clinical benefit, QoL, and safety profile [195].

Recently, a new and very efficient CDK4/6–RB1 axis blocker has emerged as an antitumor drug thanks to its interesting efficacy in breast and ovarian cancers, among other malignant neoplasms [196]. The SPH3643 molecule displayed a stronger inhibition pattern and better specificity than abemaciclib and palbociclib, opening a new window for future CTs focused on the development of specific strategies in RB1-positive cancers.

## 4. Discussion

Obesity is a leading cause and a risk factor in cancer development. Bad dietary habits and high BMI are estimated to account for 15–20% of all cancer-related deaths and joined to physical inactivity, these three preventable factors contribute to around 20 to 30% of the most commonly diagnosed malignancies. DIO has been seen to interfere with several cellular and molecular mechanisms that set off the bases for tumor development. Indeed, the present research is focused on the understanding of the molecular mechanisms favoring cancer development in obese patients, as well as the underlying pathways that unleash the inflammatory state, characterized by high levels of cytokines and adipokines (IL-1, IL-6, TNF-α, leptin) and CRP, and lower concentrations of circulating APN. New elucidations within this research field could greatly contribute to improving the treatment approaches for breast and ovarian cancers, two of the most frequent female tumors with high rates of fatal outcomes.

Cyclins and CDKs are essential players that regulate cell cycle-related biological pathways. Clinical use of first-generation, pan-CDKIs such as flavopiridol or seliciclib has been replaced to avoid side effects and improve efficacy by new potent selective CDKI agents. The FDA has already approved three CDK4/6 inhibitors for lifelong breast cancer therapy (palbociclib, abemaciclib, ribociclib), which mainly work by avoiding the G1 to S cell-cycle phase transition. Interestingly, Iqbal et al., 2018 proposed CDK blockers for the management of obesity, as CDK-4 proved to be an effective target in the treatment of DIO, mostly by preventing RB1 protein from hyperphosphorylation. The RB1 unphosphorylated form allows its biological activation, which consequently reinstates POMC neuron function and leptin sensitivity, driving increased lipid peroxidation and fat mass drop.

In those terms, along with this article, we have exemplified how the next-generation CDK inhibitors targeting CDK4/6 not only represent a beneficial alternative in ovarian and breast tumors—both in monotherapy or in combination with ETs or AIs—with several CTs presently supporting this choice (monarchE, MONALEESA, NATALEE, PALLET, among others), but we have also evaluated their potential as future players in the treatment of DIO.

As has been introduced at the beginning of this article, blocking CDK proteins also induces endogenous cyclin inhibitor proteins such as p21 and p27, which are common targets of many phytochemicals and plant-based foods such as those mentioned in this review (p.e. curcumin, vitamins A, C, E; anthocyanins…), that further decrease RB1 phosphorylation and consequently avoid tumor cells invasion, migration, and angiogenesis. Regarding the potential introduction of CDKi drugs for the management of obesity, it would be important to estimate the anti-DIO effect in several cohorts of patients treated with CDK4/6 antagonists, as well as to identify which biomarkers could be the most useful to detect and quantify the connection existing between the anti-obesogenic role of a specific molecule and the achieved antitumoral effect. An example of these biomarkers linking both diseases has been recently reviewed last year [11], where the authors have deeply examined the accumulation of reactive oxygen species (ROS) and other free radicals responsible for causing DNA damage in DIO and cancer, some of them already outlined in this review (p.e. adipokines and cytokines). High levels of these molecules can generate cellular DNA damage, altering additional biomolecules throughout the cell that subsequently affect the normal function of lipids and proteins as well.

The second aim of this review was to delve into the meaningful anticancer properties that dietary approaches and nutraceuticals have in the management of breast and ovarian tumors, including a better clinical response to chemotherapy and a significant reduction of adverse drug events. Several CTs have recently brought into light the likeliness of introducing nutritional and plant-based supplements in a regular diet to lower the risk of suffering or ameliorate the course of a pathological condition, by decreasing cancer growth-related factors and increasing PFS and prognosis. These studies highlight the role of phytochemicals as agents that can not only prevent but also treat chronic diseases. These new diet-based alternatives have been proposed as good tools that could be synergistically employed together with CDKIs to increase their effectiveness in breast and ovarian cancers, by targeting CDK-related proteins.

In summary, the molecular mechanisms linking obesity and cancer are getting profound interest, and many studies devoted to understanding the impact of the metabolic inflammatory state associated with obesity upon cell-cycle progression and tumor proliferation—two of the main hallmarks of cancer—are still ongoing, with a novel focus on blocking the CDK4/6 protein.

Nonetheless, several limitations need to be overcome so as to include in the treatment guidelines for cancer and obesity all the beneficial outcomes that nutraceuticals have been seen to exert both in vitro and in CTs.

One of the main difficulties of testing phytochemicals in vivo that may also hamper more accurate CTs is the quantitation and identification of which specific phytochemical or compound—usually administered as a mix of active ingredients—comes effectively absorbed into the bloodstream, especially when administered orally. Indeed, inter-individual variabilities and phytonutrient’s metabolic differences among several groups may increase the arduousness in making these molecules representative of a regular diet; this issue could additionally become more insidious if we consider that the extract’s richness in a specific metabolite can be very variable according to the extraction method of natural compounds from the plant, their purification, conservation, and climatic conditions, specific variety or specie, etc., which may further mislead the identification of the most active compound within a complex plant extract mixture of bioactive forms that can or not act synergistically.

A second hurdle in carrying out clinical studies with nutritional compounds is the lack of accurate methods that help to understand the molecular mechanisms underlying the anticancer and/or anti-obesogenic effects of these dietary phytonutrients. It is extremely complicated to design clinical studies that precisely assess the impact of a specific kind of diet on the expression of particular genes of interest, which would make it easy to predict a better treatment response within these patients. Furthermore, the majority of the studies involving nutritional approaches in breast and ovarian cancers that have been reviewed here (see Table 2) are early Phase I/II CTs, which, joined to the huge heterogeneousness among different tumor types in terms of grade, tissue, invasiveness, or genes involved, make the achievement of a common and general conclusion even more difficult.

Contrastingly, many of the studies cited in this review showed the advantages of using nutraceuticals in cancer patients, particularly those affected by obesity. These promising metabolites could become good agents to specifically increase the sensitization of tumor cells to standard chemotherapeutic drugs such as tamoxifen, paclitaxel, carboplatin, or bevacizumab—among others—and reduce different types of breast and ovarian cancers. Furthermore, the synergistic effect of chemotherapy and phytonutrients may be of great help to diminish chemoresistance and/or the toxicity typically related to these drugs, improving the prognoses of diagnosed cancers. Thus, it is important to further investigate the use of phytochemicals in future CTs, as well as to extend their applications to other human malignancies, to deduct to what extent they may effectively become a potent long-term strategy.

A future challenge consists in the development of novel personalized medicine to treat obesity and related diseases, repurposing some already commercialized cancer-associated therapeutic strategies such as CDK4/6 inhibitors or CDK druggable proteins that have been presented along with this review. Furthermore, the potential combination of nutraceuticals and/or dietary supplements with CDKi could open a new research pathway to be further exploited in the cancer research field. As a matter of fact, a recently published work has also shown Camptothecin—an anticancer-proved molecule targeting topoisomerase enzymes—to be effective in treating DIO in vivo, probably throughout the activation of the growth differentiation factor GDF15, therefore decreasing BMI, blood glucose, and hepatic fatness [197]. This newfound article represents another example of the increasing interest that antitumor agents may have in the management of obesity, linking cancer mechanisms with those underlying DIO development, which undoubtedly need further investigation and CTs set up to effectively understand their efficacy and safety of use.

## 5. Conclusions

The strong correlation linking obesity and cancer has been widely investigated and ascertained among the last years. Nonetheless, the specific pathways that may help to understand this essential association have not been completely deciphered. Along this review we have highlighted the specific and potential mechanistic similarities conecting both kind of diseases, underlining the outcomes reached by several related and very recent studies and clinical trials, with a special focus given to cyclins and CDKs. We have also emphasized the anticancer properties of plant-derived compounds and nutraceuticals that are frequently found in the Mediterranean diet, which may represent a safe and beneficial option that complement the antitumor therapy. 

In conclusion, the data here summarized support the fact that DIO and cancer are two tightly connected conditions, and CDK-pathways represent a key molecular link between breast and ovarian malignancies with obesity. We hope that this review may contribute to promote additional research that explore the promising properties of CDKIs as valuable anticancer agents in obese patients and viceversa.

## Figures and Tables

**Table 3:** Selected randomized and ongoing clinical trials evaluating CDKIs therapy in advanced breast and ovarian cancers.

CDKI	Target	Outcome	N	Disease	Phase	Study
PD-0332991 Palbociclib	CDK 4/6	Palbociclib efficacy and safety were confirmed in recurrent ovarian cancer. Biochemical response rate was determined by CA125	26	Ovarian epithelial carcinoma	Phase II	[157]
Palbociclib	CDK 4/6	Palbociclib monotherapy is effective and well tolerated in endocrine-resistant HR+/Rb+ breast cancer patients. Treatment-related neutropenia was significantly associated with extended PFS, suggesting neutropenia as a useful marker to set up palbociclib dosing	196	(1) Metastatic breast cancer (MBC) (2) Metastatic colorectal cancer (3) Metastatic melanoma with CDK4 mutation or amplification (4) Cisplatin-refractory, unresectable germ cell tumors	Phase II	[158,167]
Palbociclib	CDK 4/6	Palbociclib plus endocrine therapy (Letrozole; PALOMA-2; or Fulvestrant, PALOMA-3) showed significant and extended efficacy and good drug tolerance regardless of molecular cancer subtype. CDKI 4/6 median PFS was twice vs. endocrine monotherapy	666	ER+/Her2- advanced breast cancer (ABC)	Phase III	PALOMA [156,168,169]
Palbociclib	CDK 4/6	Palbociclib exhibited promising clinical activity in monotherapy and may become a potential strategy to overcome resistance in patients with prior response to ET The median clinical benefit was two times higher when combined with HT (anastrozole, letrozole, exemestane, fulvestrant) vs. palbociclib alone	115	ER+/Her2- postmenopausal ABC	Phase II	TREnd trial [155]
Palbociclib	CDK 4/6	Palbociclib plus letrozole therapy decreased BC cell proliferation and induced a full cell-cycle block in ER+ patients compared with the letrozole group. Slight neutropenia levels were observed	306	ER+ primary breast cancer	Phase II	PALLET [170]
LY2835219 Abemaciclib	CDK 4/6	Abemaciclib monotherapy exhibited promising clinical activity and safety in refractory HR+/Her2- MBC patients.Extended CT also confirmed good biological effects of abemaciclib in co-treatment with anastrozole	224 132	HR+/Her2- metastatic breast cancer	Phase II	MONARCH-1 [165,171]
Abemaciclib	CDK 4/6	PFS improvement for patients receiving abemaciclib + fulvestrant or fulvestrant monotherapy regardless of menopausal status. The combined treatment was safe and effective, significantly delaying successive chemotherapy cycles	669	HR+/HER2- ABC	Phase III	MONARCH-2 [172,173]
Abemaciclib	CDK 4/6	Abemaciclib plus ET significantly ameliorated IDFS in patients at high risk of recurrence compared with ET monotherapy	5637	HR+, Her2-, node-positive early breast cancer	Phase III	monarchE [174,175]
Abemaciclib	CDK 4/6	Good safety profile and efficacy in combinations of abemaciclib with different ET in HR+, HER2- MBC patients. Evidence of antitumor activity: ORR of 38.9% and CBR of 55.6% First study that evaluates anticancer effect of abemaciclib + exemestane	37	Rb+, triple negative MBC	Phase Ib	[176]
Abemaciclib	CDK 4/6	Estimation of the PFS and clinical benefit rate Patients with HR+ tumors also receive AIs (anastrozole/letrozole) for standard of care (SOC)	32	Recurrent ovarian and endometrial cancer	Phase II	[177]
LEE011 Ribociclib	CDK 4/6	Ribociclib showed clinical activity in some tumor types The association between the genomic mutation profile and the observed clinical benefits is still under study, in order to assess the co-treatment potential with additional drugs	106	Tumors with CDK4/6 pathway activation	Phase II	[178]
Ribociclib	CDK 4/6	The addition of ribociclib to letrozole treatment (low clinical activity as a single agent) generated 50% and 55% PFS in patients with ER+ relapsed OC and EC, respectively	40	Ovarian and endometrial cancer	Phase II	[179,180]
Ribociclib	CDK 4/6	The co-treatment of ribociclib and fulvestrant exerted a significant overall survival benefit vs. placebo and fulvestrant in HR+/Her2-ABC patients, opening a new first/second-line of treatment for this cancer subtype	725	ER+ breast cancer	Phase III	MONALEESA-3 [181,182]
Ribociclib	CDK 4/6	Ribociclib plus multiple first-line ETs (tamoxifen/NSAI + goserelin) significantly increased PFS and showed a moderated toxicity profile	672	HR+/Her2- advanced breast cancer	Phase III	MONALEESA-7 [183]
Ribociclib	CDK 4/6	To understand the ability of Ribociclib plus ET to prevent or delay acquired resistance to ET in BC patients, so as to improve IDFS	5000	HR+/Her2- early breast cancer	Phase III	NATALEE [184]
G1T28 Trilaciclib	CDK 4/6	Prevention of chemotherapy- induced myelosuppression in TNBC is being tested Low-toxicity regimen generally well tolerated exerting encouraging survival rates No treatment-related deaths were identified	102	Triple negative breast cancer	Phase II	[185]
SY-1365	CDK 7	SY-1365 inhibited cancer cell growth in vitro and murine xenograft models Enhanced activity was observed in combinations with BCL2 inhibitor (venetoclax)	137	Ovarian cancer breast cancer	Phase I	First selective CDK7 inhibitor to enter clinical development [144]

ABC: advanced breast cancer, AI: aromatase inhibitor, CBR: clinical benefit rate, CT: chemotherapy, EBC: early breast cancer, EC: endometrial cancer, ER: estrogen receptor, ET/HT: endocrine/hormonal therapy; HR: hormonal receptor, IDFS: invasive disease-free survival, MBC: metastatic breast cancer, OC: ovarian cancer, ORR: objective response rate, OS: overall survival PFS: progression-free survival, SOC: standard of care, TNBC: triple-negative breast cancer.

## References

[1] World Health Organization Global Health Estimates: Life Expectancy and Leading Causes of Death and Disability. *The Global Health Observatory*, 2019. https://www.who.int/data/gho/data/themes/mortality-and-global-health-estimates.

[2] Wang H., Abbas K.M., Abbasifard M., Abbasi-Kangevari M., Abbastabar H., Abd-Allah F., Abdelalim A., Abolhassani H., Abreu L.G., Abrigo M.R.M. (2020). Global age-sex-specific fertility, mortality, healthy life expectancy (HALE), and population estimates in 204 countries and territories, 1950–2019: A comprehensive demographic analysis for the Global Burden of Disease Study 2019. Lancet.

[3] Siegel R.L., Miller K.D., Fuchs H.E., Jemal A. (2021). Cancer Statistics, 2021. CA Cancer J. Clin..

[4] Giordano A., Pinto C., Pentimalli F. (2021). Cancer research and care in the future post COVID 19 era. Ann. Res. Oncol..

[5] World Health Organization WHO Methods and Data Sources for Country-Level Causes of Death 2000–2016. *World Health Organization*, 2018. https://www.who.int/docs/default-source/gho-documents/global-health-estimates/ghe2019_cod_methods.pdf.

[6] Kabat G.C., Matthews C.E., Kamensky V., Hollenbeck A.R., Rohan T.E. (2015). Adherence to cancer prevention guidelines and cancer incidence, cancer mortality, and total mortality: A prospective cohort study. Am. J. Clin. Nutr..

[7] Grosso G., Bella F., Godos J., Sciacca S., Del Rio D., Ray S., Galvano F., Giovannucci E.L. (2017). Possible role of diet in cancer: Systematic review and multiple meta-analyses of dietary patterns, lifestyle factors, and cancer risk. Nutr. Rev..

[8] International Agency for Research on Cancer (IARC)—World Health Organization (2002). IARC Handbooks of Cancer Prevention: Weight Control and Physical Activity.

[9] World Cancer Research Fund/American Institute for Cancer Research Diet, Nutrition, Physical Activity and Cancer: A Global Perspective. Continuous Update Project Expert Report 2018. https://www.wcrf.org/diet-activity-and-cancer/.

[10] McCubrey J.A., Lertpiriyapong K., Steelman L.S., Abrams S.L., Yang L.V., Murata R.M., Rosalen P.L., Scalisi A., Neri L.M., Cocco L. (2017). Effects of resveratrol, curcumin, berberine and other nutraceuticals on aging, cancer development, cancer stem cells and microRNAs. Aging.

[11] Holm J.B., Rosendahl A.H., Borgquist S. (2021). Local Biomarkers Involved in the Interplay between Obesity and Breast Cancer. Cancers.

[12] Tanha K., Mottaghi A., Nojomi M., Moradi M., Rajabzadeh R., Lotfi S., Janani L. (2021). Investigation on factors associated with ovarian cancer: An umbrella review of systematic review and meta-analyses. J. Ovarian Res..

[13] Bray F., Ferlay J., Soerjomataram I., Siegel R.L., Torre L.A., Jemal A. (2018). Global cancer statistics 2018: GLOBOCAN estimates of incidence and mortality worldwide for 36 cancers in 185 countries. CA Cancer J. Clin..

[14] American Cancer Society (2021). American Cancer Society: Cancer Facts and Figures 2021.

[15] American Cancer Society (2020). American Cancer Society. Cancer Facts and Figures 2020.

[16] Howlader N., Noone A.M., Krapcho M., Miller D., Brest A., Yu M., Ruhl J., Tatalovich Z., Mariotto A., Lewis D.R. SEER Cancer Statistics Review, 1975–2018, National Cancer Institute. Bethesda, MD. Based on November 2020 SEER Data Submission, Posted to the SEER Web Site, April 2021. https://seer.cancer.gov/csr/1975_2018/.

[17] Park J., Morley T.S., Kim M., Clegg D.J., Scherer P.E. (2014). Obesity and cancer—Mechanisms underlying tumour progression and recurrence. Nat. Rev. Endocrinol..

[18] Khandekar M.J., Cohen P., Spiegelman B.M. (2011). Molecular mechanisms of cancer development in obesity. Nat. Rev. Cancer.

[19] Peng H., Wang Q., Qi X., Wang X., Zhao X. (2018). Orlistat induces apoptosis and protective autophagy in ovarian cancer cells: Involvement of Akt-mTOR-mediated signaling pathway. Arch. Gynecol. Obstet..

[20] Menendez J.A., Vellon L., Lupu R. (2006). The antiobesity drug Orlistat induces cytotoxic effects, suppresses Her-2/neu (erbB-2) oncogene overexpression, and synergistically interacts with trastuzumab (Herceptin^TM^) in chemoresistant ovarian cancer cells. Int. J. Gynecol. Cancer.

[21] Xiao X., Liu H., Li X. (2017). Orlistat treatment induces apoptosis and arrests cell cycle in HSC-3 oral cancer cells. Microb. Pathog..

[22] Iqbal N.J., Lu Z., Liu S.M., Schwartz G.J., Chua S., Zhu L. (2018). Cyclin-dependent kinase 4 is a preclinical target for diet-induced obesity. JCI Insight.

[23] Iqbal N., Zhu L., Chua S.C. (2020). Neuronal Cell Cycle Events Link Caloric Intake to Obesity. Trends Endocrinol. Metab..

[24] Noonan J.J., Jarzabek M., Lincoln F.A., Cavanagh B.L., Pariag A.R., Juric V., Young L.S., Ligon K.L., Jahns H., Zheleva D. (2019). Implementing Patient-Derived Xenografts to Assess the Effectiveness of Cyclin-Dependent Kinase Inhibitors in Glioblastoma. Cancers.

[25] Law M.E., Corsino P.E., Narayan S., Law B.K. (2015). Cyclin-Dependent Kinase Inhibitors as Anticancer Therapeutics. Mol. Pharmacol..

[26] Juric V., Hudson L., Fay J., Richards C.E., Jahns H., Verreault M., Bielle F., Idbaih A., Lamfers M.L.M., Hopkins A.M. (2021). Transcriptional CDK inhibitors, CYC065 and THZ1 promote Bim-dependent apoptosis in primary and recurrent GBM through cell cycle arrest and Mcl-1 downregulation. Cell Death Dis..

[27] Zhou Q. (2017). Targeting Cyclin-Dependent Kinases in Ovarian Cancer. Cancer Investig..

[28] Blücher C., Stadler S.C. (2017). Obesity and Breast Cancer: Current Insights on the Role of Fatty Acids and Lipid Metabolism in Promoting Breast Cancer Growth and Progression. Front. Endocrinol..

[29] Wu Q., Li B., Li Z., Li J., Sun S., Sun S. (2019). Cancer-associated adipocytes: Key players in breast cancer progression. J. Hematol. Oncol..

[30] Olsen C.M., Nagle C.M., Whiteman D.C., Ness R., Pearce C.L., Pike M.C., Rossing M.A., Terry K.L., Wu A.H., A Risch H. (2013). Obesity and risk of ovarian cancer subtypes: Evidence from the Ovarian Cancer Association Consortium. Endocr. Relat. Cancer.

[31] Howe L.R., Subbaramaiah K., Hudis C.A., Dannenberg A.J. (2013). Molecular Pathways: Adipose Inflammation as a Mediator of Obesity-Associated Cancer. Clin. Cancer Res..

[32] Kwon O., Kim K.W., Kim M.S. (2016). Leptin signalling pathways in hypothalamic neurons. Cell. Mol. Life Sci..

[33] Ptak A., Kolaczkowska E., Gregoraszczuk E.L. (2013). Leptin stimulation of cell cycle and inhibition of apoptosis gene and protein expression in OVCAR-3 ovarian cancer cells. Endocrine.

[34] Newman G., Gonzalez-Perez R.R. (2014). Leptin–cytokine crosstalk in breast cancer. Mol. Cell. Endocrinol..

[35] Carpenter B., Hemsworth G.R., Wu Z., Maamra M., Strasburger C.J., Ross R.J., Artymiuk P.J. (2012). Structure of the Human Obesity Receptor Leptin-Binding Domain Reveals the Mechanism of Leptin Antagonism by a Monoclonal Antibody. Structure.

[36] Catalano S., Leggio A., Barone I., De Marco R., Gelsomino L., Campana A., Malivindi R., Panza S., Giordano C., Liguori A. (2015). A novel leptin antagonist peptide inhibits breast cancer growth in vitro and in vivo. J. Cell. Mol. Med..

[37] Catalano S., Mauro L., Bonofiglio D., Pellegrino M., Qi H., Rizza P., Vizza D., Bossi G., Andò S. (2011). In Vivo and in Vitro Evidence That PPARγ Ligands Are Antagonists of Leptin Signaling in Breast Cancer. Am. J. Pathol..

[38] Atoum M.F., Alzoughool F., Al-Hourani H. (2020). Linkage Between Obesity Leptin and Breast Cancer. Breast Cancer Basic Clin. Res..

[39] Otvos L., Kovalszky I., Riolfi M., Ferla R., Olah J., Sztodola A., Nama K., Molino A., Piubello Q., Wade J.D. (2011). Efficacy of a leptin receptor antagonist peptide in a mouse model of triple-negative breast cancer. Eur. J. Cancer.

[40] Lipsey C.C., Harbuzariu A., Daley-Brown D., Gonzalez-Perez R.R. (2016). Oncogenic role of leptin and Notch interleukin-1 leptin crosstalk outcome in cancer. World J. Methodol..

[41] Harmon T., Harbuzariu A., Yang L., Gonzalez-Perez R.R. (2014). 267 Novel adjuvant therapy with leptin peptide receptor antagonist-2 conjugated to nanoparticles (IONP-LPrA2) to minimize chemoresistance in triple negative breast cancer. Eur. J. Cancer.

[42] Giordano C., Chemi F., Panza S., Barone I., Bonofiglio D., Lanzino M., Cordella A., Campana A., Hashim A., Rizza P. (2016). Leptin as a mediator of tumor-stromal interactions promotes breast cancer stem cell activity. Oncotarget.

[43] Barone I., Giordano C., Bonofiglio D., Andò S., Catalano S. (2016). Leptin, obesity and breast cancer: Progress to understanding the molecular connections. Curr. Opin. Pharmacol..

[44] Bowers L.W., Rossi E.L., McDonell S.B., Doerstling S.S., Khatib S.A., Lineberger C.G., Albright J.E., Tang X., Degraffenried L.A., Hursting S.D. (2018). Leptin Signaling Mediates Obesity-Associated CSC Enrichment and EMT in Preclinical TNBC Models. Mol. Cancer Res..

[45] Lane D., Matte I., Garde-Granger P., Laplante C., Carignan A., Rancourt C., Piché A. (2015). Inflammation-regulating factors in ascites as predictive biomarkers of drug resistance and progression-free survival in serous epithelial ovarian cancers. BMC Cancer.

[46] Crean-Tate K.K., Reizes O. (2018). Leptin Regulation of Cancer Stem Cells in Breast and Gynecologic Cancer. Endocrinology.

[47] Jarde T., Caldefie-Chézet F., Damez M., Mishellany F., Perrone D., Penault-Llorca F., Guillot J., Vasson M.P. (2008). Adiponectin and leptin expression in primary ductal breast cancer and in adjacent healthy epithelial and myoepithelial tissue. Histopathology.

[48] Zheng Q., Dunlap S.M., Zhu J., Downs-Kelly E., Rich J., Hursting S.D., Berger N.A., Reizes O. (2011). Leptin deficiency suppresses MMTV-Wnt-1 mammary tumor growth in obese mice and abrogates tumor initiating cell survival. Endocr. Relat. Cancer.

[49] Chang C.C., Wu M.J., Yang J.Y., Camarillo I.G., Chang C.J. (2015). Leptin–STAT3–G9a Signaling Promotes Obesity-Mediated Breast Cancer Progression. Cancer Res..

[50] Wang T., Fahrmann J.F., Lee H., Li Y.-J., Tripathi S.C., Yue C., Zhang C., Lifshitz V., Song J., Yuan Y. (2018). JAK/STAT3-Regulated Fatty Acid β-Oxidation Is Critical for Breast Cancer Stem Cell Self-Renewal and Chemoresistance. Cell Metab..

[51] Di Zazzo E., Feola A., Zuchegna C., Romano A., Donini C.F., Bartollino S., Costagliola C., Frunzio R., Laccetti P., Di Domenico M. (2014). The p85 Regulatory Subunit of PI3K Mediates cAMP-PKA and Insulin Biological Effects on MCF-7 Cell Growth and Motility. Sci. World J..

[52] Di Zazzo E., Polito R., Bartollino S., Nigro E., Porcile C., Bianco A., Daniele A., Moncharmont B. (2019). Adiponectin as Link Factor between Adipose Tissue and Cancer. Int. J. Mol. Sci..

[53] Katira A., Tan P.H. (2016). Evolving role of adiponectin in cancer-controversies and update. Cancer Biol. Med..

[54] Mauro L., Pellegrino M., De Amicis F., Ricchio E., Giordano F., Rizza P., Catalano S., Bonofiglio D., Sisci D., Panno M.L. (2014). Evidences that estrogen receptor α interferes with adiponectin effects on breast cancer cell growth. Cell Cycle.

[55] Dieudonne M.N., Bussiere M., dos Santos E., Leneveu M.C., Giudicelli Y., Pecquery R. (2006). Adiponectin mediates antiproliferative and apoptotic responses in human MCF7 breast cancer cells. Biochem. Biophys. Res. Commun..

[56] Diaz E.S., Karlan B.Y., Li A.J. (2013). Obesity-associated adipokines correlate with survival in epithelial ovarian cancer. Gynecol. Oncol..

[57] Lawrence T. (2007). Inflammation and cancer: A failure of resolution?. Trends Pharmacol. Sci..

[58] Lan T., Chen L., Wei X. (2021). Inflammatory Cytokines in Cancer: Comprehensive Understanding and Clinical Progress in Gene Therapy. Cells.

[59] Donovan J., Slingerland J. (2000). Transforming growth factor-β and breast cancer: Cell cycle arrest by transforming growth factor-β and its disruption in cancer. Breast Cancer Res..

[60] Batlle E., Massagué J. (2019). Transforming Growth Factor-β Signaling in Immunity and Cancer. Immunity.

[61] Murugaiyan G., Saha B. (2009). Protumor vs Antitumor Functions of IL-17. J. Immunol..

[62] Eiró N., González L., González L.O., Fernandez-Garcia B., Lamelas M.L., Marín L., González-Reyes S., del Casar J.M., Vizoso F.J. (2012). Relationship between the Inflammatory Molecular Profile of Breast Carcinomas and Distant Metastasis Development. PLoS ONE.

[63] Macciò A., Madeddu C., Gramignano G., Mulas C., Floris C., Massa D., Astara G., Chessa P., Mantovani G. (2010). Correlation of body mass index and leptin with tumor size and stage of disease in hormone-dependent postmenopausal breast cancer: Preliminary results and therapeutic implications. J. Mol. Med..

[64] Pereira J.F.S., Bessa C., Matos P., Jordan P. (2022). Pro-Inflammatory Cytokines Trigger the Overexpression of Tumour-Related Splice Variant RAC1B in Polarized Colorectal Cells. Cancers.

[65] Browning L., Patel M.R., Horvath E.B., Tawara K., Jorcyk C.L. (2018). IL-6 and ovarian cancer: Inflammatory cytokines in promotion of metastasis. Cancer Manag. Res..

[66] Deng F., Weng Y., Li X., Wang T., Fan M., Shi Q. (2020). Overexpression of IL-8 promotes cell migration via PI3K-Akt signaling pathway and EMT in triple-negative breast cancer. Pathol. Res. Pract..

[67] Li Z., Jiang J., Wang Z., Zhang J., Xiao M., Wang C., Lu Y., Qin Z. (2008). Endogenous Interleukin-4 Promotes Tumor Development by Increasing Tumor Cell Resistance to Apoptosis. Cancer Res..

[68] Heim L., Yang Z., Tausche P., Hohenberger K., Chiriac M.T., Koelle J., Geppert C.-I., Kachler K., Miksch S., Graser A. (2022). IL-9 Producing Tumor-Infiltrating Lymphocytes and Treg Subsets Drive Immune Escape of Tumor Cells in Non-Small Cell Lung Cancer. Front. Immunol..

[69] Toney N.J., Opdenaker L.M., Cicek K., Frerichs L., Kennington C.R., Oberly S., Archinal H., Somasundaram R., Sims-Mourtada J. (2022). Tumor-B-cell interactions promote isotype switching to an immunosuppressive IgG4 antibody response through upregulation of IL-10 in triple negative breast cancers. J. Transl. Med..

[70] Ahmad N., Ammar A., Storr S.J., Green A.R., Rakha E., Ellis I.O., Martin S.G. (2018). IL-6 and IL-10 are associated with good prognosis in early stage invasive breast cancer patients. Cancer Immunol. Immunother..

[71] Itakura E., Huang R.R., Wen D.R., Paul E., Wünsch P.H., Cochran A.J. (2011). IL-10 expression by primary tumor cells correlates with melanoma progression from radial to vertical growth phase and development of metastatic competence. Mod. Pathol..

[72] Zhao S., Wu D., Wu P., Wang Z., Huang J., Gao J.X. (2015). Serum IL-10 Predicts Worse Outcome in Cancer Patients: A Meta-Analysis. PLoS ONE.

[73] Liubomirski Y., Lerrer S., Meshel T., Morein D., Rubinstein-Achiasaf L., Sprinzak D., Wiemann S., Körner C., Ehrlich M., Ben-Baruch A. (2019). Notch-Mediated Tumor-Stroma-Inflammation Networks Promote Invasive Properties and CXCL8 Expression in Triple-Negative Breast Cancer. Front. Immunol..

[74] Liubomirski Y., Ben-Baruch A. (2020). Notch-Inflammation Networks in Regulation of Breast Cancer Progression. Cells.

[75] Al-Hatamleh M.A.I., Ahmad S., Boer J., Lim J., Chen X., Plebanski M., Mohamud R. (2019). A Perspective Review on the Role of Nanomedicine in the Modulation of TNF-TNFR2 Axis in Breast Cancer Immunotherapy. J. Oncol..

[76] Gubernatorova E., Polinova A., Petropavlovskiy M., Namakanova O., Medvedovskaya A., Zvartsev R., Telegin G., Drutskaya M., Nedospasov S. (2021). Dual Role of TNF and LTα in Carcinogenesis as Implicated by Studies in Mice. Cancers.

[77] Cruceriu D., Baldasici O., Balacescu O., Berindan-Neagoe I. (2020). The dual role of tumor necrosis factor-alpha (TNF-α) in breast cancer: Molecular insights and therapeutic approaches. Cell. Oncol..

[78] Westrich J.A., Vermeer D.W., Colbert P.L., Spanos W.C., Pyeon D. (2020). The multifarious roles of the chemokine CXCL14 in cancer progression and immune responses. Mol. Carcinog..

[79] Saahene R.O., Wang J., Wang M.L., Agbo E., Song H. (2019). The role of CXC chemokine ligand 4/CXC chemokine receptor 3-B in breast cancer progression. Biotech. Histochem..

[80] Lu C., Klement J.D., Ibrahim M.L., Xiao W., Redd P.S., Nayak-Kapoor A., Zhou G., Liu K. (2019). Type I interferon suppresses tumor growth through activating the STAT3-granzyme B pathway in tumor-infiltrating cytotoxic T lymphocytes. J. Immunother. Cancer.

[81] Heichler C., Scheibe K., Schmied A., Geppert C.I., Schmid B., Wirtz S., Thoma O.-M., Kramer V., Waldner M.J., Büttner C. (2020). STAT3 activation through IL-6/IL-11 in cancer-associated fibroblasts promotes colorectal tumour development and correlates with poor prognosis. Gut.

[82] Ke W., Zhang L., Dai Y. (2020). The role of IL-6 in immunotherapy of non-small cell lung cancer (NSCLC) with immune-related adverse events (irAEs). Thorac. Cancer.

[83] Wan J., Wu Y., Ji X., Huang L., Cai W., Su Z., Wang S., Xu H. (2020). IL-9 and IL-9-producing cells in tumor immunity. Cell Commun. Signal..

[84] Marusyk A., Tabassum D.P., Altrock P.M., Almendro V., Michor F., Polyak K. (2014). Non-cell-autonomous driving of tumour growth supports sub-clonal heterogeneity. Nature.

[85] Zhao J., Chen X., Herjan T., Li X. (2020). The role of interleukin-17 in tumor development and progression. J. Exp. Med..

[86] Suarez-Carmona M., Lesage J., Cataldo D., Gilles C. (2017). EMT and inflammation: Inseparable actors of cancer progression. Mol. Oncol..

[87] Jo E., Jang H.-J., Yang K.E., Jang M.S., Huh Y.H., Yoo H.-S., Park J.S., Jang I.-S., Park S.J. (2020). Cordyceps militaris induces apoptosis in ovarian cancer cells through TNF-α/TNFR1-mediated inhibition of NF-κB phosphorylation. BMC Complement. Med. Ther..

[88] Di Bello E., Zwergel C., Mai A., Valente S. (2020). The Innovative Potential of Statins in Cancer: New Targets for New Therapies. Front. Chem..

[89] Menendez J.A., Vellon L., Lupu R. (2005). Antitumoral actions of the anti-obesity drug orlistat (Xenical™) in breast cancer cells: Blockade of cell cycle progression, promotion of apoptotic cell death and PEA3-mediated transcriptional repression of Her2/neu (erbB-2) oncogene. Ann. Oncol..

[90] Harborg S., Heide-Jørgensen U., Ahern T.P., Ewertz M., Cronin-Fenton D., Borgquist S. (2020). Statin use and breast cancer recurrence in postmenopausal women treated with adjuvant aromatase inhibitors: A Danish population-based cohort study. Breast Cancer Res. Treat..

[91] Couttenier A., Lacroix O., Vaes E., Cardwell C.R., de Schutter H., Robert A. (2017). Statin use is associated with improved survival in ovarian cancer: A retrospective population-based study. PLoS ONE.

[92] Beckwitt C.H., Brufsky A., Oltvai Z.N., Wells A. (2018). Statin drugs to reduce breast cancer recurrence and mortality. Breast Cancer Res..

[93] Sava G.P., Fan H., Coombes R.C., Buluwela L., Ali S. (2020). CDK7 inhibitors as anticancer drugs. Cancer Metastasis Rev..

[94] Mayer E.L. (2015). Targeting Breast Cancer with CDK Inhibitors. Curr. Oncol. Rep..

[95] Tu Y., Kim E., Gao Y., Rankin G.O., Li B., Chen Y.C. (2016). Theaflavin-3, 3′-digallate induces apoptosis and G2 cell cycle arrest through the Akt/MDM2/p53 pathway in cisplatin-resistant ovarian cancer A2780/CP70 cells. Int. J. Oncol..

[96] Gorzynik-Debicka M., Przychodzen P., Cappello F., Kuban-Jankowska A., Marino Gammazza A., Knap N., Wozniak M., Gorska-Ponikowska M. (2018). Potential Health Benefits of Olive Oil and Plant Polyphenols. Int. J. Mol. Sci..

[97] Bailon-Moscoso N., Cevallos-Solorzano G., Romero-Benavides J., Ramirez Orellana M. (2017). Natural Compounds as Modulators of Cell Cycle Arrest: Application for Anticancer Chemotherapies. Curr. Genom..

[98] Woodie L.N., Johnson R.M., Ahmed B., Fowler S., Haynes W., Carmona B., Reed M., Suppiramaniam V., Greene M.W. (2020). Western diet-induced obesity disrupts the diurnal rhythmicity of hippocampal core clock gene expression in a mouse model. Brain Behav. Immun..

[99] Longo V.D., Panda S. (2016). Fasting, circadian rhythms, and time-restricted feeding in healthy lifespan. Cell Metab..

[100] Buono R., Longo V.D. (2018). Starvation, Stress Resistance, and Cancer. Trends Endocrinol. Metab..

[101] Levine M.E., Suarez J.A., Brandhorst S., Balasubramanian P., Cheng C.-W., Madia F., Fontana L., Mirisola M.G., Guevara-Aguirre J., Wan J. (2014). Low Protein Intake Is Associated with a Major Reduction in IGF-1, Cancer, and Overall Mortality in the 65 and Younger but Not Older Population. Cell Metab..

[102] Alumkal J.J., Slottke R., Schwartzman J., Cherala G., Munar M., Graff J.N., Beer T.M., Ryan C.W., Koop D.R., Gibbs A. (2015). A phase II study of sulforaphane-rich broccoli sprout extracts in men with recurrent prostate cancer. Investig. New Drugs.

[103] Amjad A.I., Parikh R.A., Appleman L.J., Hahm E.R., Singh K., Singh S.V. (2015). Broccoli-Derived Sulforaphane and Chemoprevention of Prostate Cancer: From Bench to Bedside. Curr. Pharmacol. Rep..

[104] Kim J.-H., Kwon K.H., Jung J.-Y., Han H.-S., Shim J.H., Oh S., Choi K.-H., Choi E.-S., Shin J.-A., Leem D.-H. (2010). Sulforaphane Increases Cyclin-Dependent Kinase Inhibitor, p21 Protein in Human Oral Carcinoma Cells and Nude Mouse Animal Model to Induce G2/M Cell Cycle Arrest. J. Clin. Biochem. Nutr..

[105] Justin S., Rutz J., Maxeiner S., Chun F.K.H., Juengel E., Blaheta R.A. (2020). Chronic Sulforaphane Administration Inhibits Resistance to the mTOR-Inhibitor Everolimus in Bladder Cancer Cells. Int. J. Mol. Sci..

[106] Simões B.M., Santiago-Gómez A., Chiodo C., Moreira T., Conole D., Lovell S., Alferez D., Eyre R., Spence K., Sarmiento-Castro A. (2020). Targeting STAT3 signaling using stabilised sulforaphane (SFX-01) inhibits endocrine resistant stem-like cells in ER-positive breast cancer. Oncogene.

[107] Kiechle M., Dukatz R., Yahiaoui-Doktor M., Berling A., Basrai M., Staiger V., Niederberger U., Marter N., Lammert J., Grill S. (2017). Feasibility of structured endurance training and Mediterranean diet in BRCA1 and BRCA2 mutation carriers—An interventional randomized controlled multicenter trial (LIBRE-1). BMC Cancer.

[108] Kiechle M., Engel C., Berling A., Hebestreit K., Bischoff S.C., Dukatz R., Siniatchkin M., Pfeifer K., Grill S., Yahiaoui-Doktor M. (2016). Effects of lifestyle intervention in BRCA1/2 mutation carriers on nutrition, BMI, and physical fitness (LIBRE study): Study protocol for a randomized controlled trial. Trials.

[109] Seethaler B., Basrai M., Vetter W., Lehnert K., Engel C., Siniatchkin M., Halle M., Kiechle M., Bischoff S.C. (2020). Fatty acid profiles in erythrocyte membranes following the Mediterranean diet—Data from a multicenter lifestyle intervention study in women with hereditary breast cancer (LIBRE). Clin. Nutr..

[110] Cohen C.W., Fontaine K.R., Arend R.C., Soleymani T., Gower B.A. (2018). Favorable Effects of a Ketogenic Diet on Physical Function, Perceived Energy, and Food Cravings in Women with Ovarian or Endometrial Cancer: A Randomized, Controlled Trial. Nutrients.

[111] Thomsen C.B., Andersen R.F., Steffensen K.D., Adimi P., Jakobsen A. (2019). Delta tocotrienol in recurrent ovarian cancer. A phase II trial. Pharmacol. Res..

[112] Ma Y., Chapman J., Levine M., Polireddy K., Drisko J., Chen Q. (2014). High-Dose Parenteral Ascorbate Enhanced Chemosensitivity of Ovarian Cancer and Reduced Toxicity of Chemotherapy. Sci. Transl. Med..

[113] Cerletti C., De Curtis A., Bracone F., Digesù C., Morganti A.G., Iacoviello L., De Gaetano G., Donati M.B. (2017). Dietary anthocyanins and health: Data from FLORA and ATHENA EU projects. Br. J. Clin. Pharmacol..

[114] Koppold-Liebscher D., Kessler C.S., Steckhan N., Bähr V., Kempter C., Wischnewsky M., Hübner M., Kunz B., Paul M., Zorn S. (2020). Short-term fasting accompanying chemotherapy as a supportive therapy in gynecological cancer: Protocol for a multicenter randomized controlled clinical trial. Trials.

[115] Zorn S., Ehret J., Schäuble R., Rautenberg B., Ihorst G., Bertz H., Urbain P., Raynor A. (2020). Impact of modified short-term fasting and its combination with a fasting supportive diet during chemotherapy on the incidence and severity of chemotherapy-induced toxicities in cancer patients—A controlled cross-over pilot study. BMC Cancer.

[116] De Groot S., Lugtenberg R.T., Cohen D., Welters M.J., Ehsan I., Vreeswijk M.P., Smit V.T., de Graaf H., Heijns J.B., Portielje J.E. (2020). Fasting mimicking dide as an adjunct to neoadjuvant chemotherapy for breast cancer in the multicentre randomized phase 2 DIRECT trial. Nat. Commun..

[117] Lugtenberg R.T., de Groot S., Kaptein A.A., Fischer M.J., Kranenbarg E.M.-K., Carpentier M.D.-D., Cohen D., de Graaf H., Heijns J.B., on behalf of the Dutch Breast Cancer Research Group (BOOG) (2020). Quality of life and illness perceptions in patients with breast cancer using a fasting mimicking diet as an adjunct to neoadjuvant chemotherapy in the phase 2 DIRECT (BOOG 2013–14) trial. Breast Cancer Res. Treat..

[118] Ávila-Gálvez M.Á., García-Villalba R., Martínez-Díaz F., Ocaña-Castillo B., Monedero-Saiz T., Torrecillas-Sánchez A., Abellán B., González-Sarrías A., Espín J.C. (2019). Metabolic Profiling of Dietary Polyphenols and Methylxanthines in Normal and Malignant Mammary Tissues from Breast Cancer Patients. Mol. Nutr. Food Res..

[119] Ávila-Gálvez M., González-Sarrías A., Martínez-Díaz F., Abellán B., Martínez-Torrano A.J., Fernández-López A.J., Giménez-Bastida J.A., Espín J.C. (2021). Disposition of Dietary Polyphenols in Breast Cancer Patients’ Tumors, and Their Associated Anticancer Activity: The Particular Case of Curcumin. Mol. Nutr. Food Res..

[120] Saghatelyan T., Tananyan A., Janoyan N., Tadevosyan A., Petrosyan H., Hovhannisyan A., Hayrapetyan L., Arustamyan M., Arnhold J., Rotmann A.-R. (2020). Efficacy and safety of curcumin in combination with paclitaxel in patients with advanced, metastatic breast cancer: A comparative, randomized, double-blind, placebo-controlled clinical trial. Phytomedicine.

[121] Cover C.M., Hsieh S.J., Tran S.H., Hallden G., Kim G.S., Bjeldanes L.F., Firestone G.L. (1998). Indole-3-carbinol Inhibits the Expression of Cyclin-dependent Kinase-6 and Induces a G1 Cell Cycle Arrest of Human Breast Cancer Cells Independent of Estrogen Receptor Signaling. J. Biol. Chem..

[122] Aggarwal B.B., Ichikawa H. (2005). Molecular Targets and Anticancer Potential of Indole-3-Carbinol and Its Derivatives. Cell Cycle.

[123] Wang H., Liu L., Lin J.Z., Aprahamian T.R., Farmer S.R. (2016). Browning of White Adipose Tissue with Roscovitine Induces a Distinct Population of UCP1 + Adipocytes. Cell Metab..

[124] Wȩsierska-Ga̧dek J., Gritsch D., Zulehner N., Komina O., Maurer M. (2011). Interference with ER-α enhances the therapeutic efficacy of the selective CDK inhibitor roscovitine towards ER-positive breast cancer cells. J. Cell. Biochem..

[125] Xing Z., Zhang Y., Zhang X., Yang Y., Ma Y., Pang D. (2013). Fangchinoline Induces G1 Arrest in Breast Cancer Cells Through Cell-Cycle Regulation. Phytotherapy Res..

[126] Kim J., Yu J.-H., Ko E., Lee K.-W., Song A., Park S., Shin I., Han W., Noh D. (2010). The alkaloid Berberine inhibits the growth of Anoikis-resistant MCF-7 and MDA-MB-231 breast cancer cell lines by inducing cell cycle arrest. Phytomedicine.

[127] Chen Q., Qin R., Fang Y., Li H. (2015). Berberine Sensitizes Human Ovarian Cancer Cells to Cisplatin Through miR-93/PTEN/Akt Signaling Pathway. Cell. Physiol. Biochem..

[128] Jin P., Zhang C., Li N. (2015). Berberine Exhibits Antitumor Effects in Human Ovarian Cancer Cells. Anti-Cancer Agents Med. Chem..

[129] Cao L., Yang Y., Ye Z., Lin B., Zeng J., Li C., Liang T., Zhou K., Li J. (2018). Quercetin-3-methyl ether suppresses human breast cancer stem cell formation by inhibiting the Notch1 and PI3K/Akt signaling pathways. Int. J. Mol. Med..

[130] Li J., Zhu F., Lubet R.A., De Luca A., Grubbs C., Ericson M.E., D'Alessio A., Normanno N., Dong Z., Bode A.M. (2013). Quercetin-3-methyl ether inhibits lapatinib-sensitive and -resistant breast cancer cell growth by inducing G2 /M arrest and apoptosis. Mol. Carcinog..

[131] Meeran S.M., Katiyar S.K. (2008). Cell cycle control as a basis for cancer chemoprevention through dietary agents. Front. Biosci..

[132] Giordano A., Tommonaro G. (2019). Curcumin and Cancer. Nutrients.

[133] Hu S., Xu Y., Meng L., Huang L., Sun H. (2018). Curcumin inhibits proliferation and promotes apoptosis of breast cancer cells. Exp. Ther. Med..

[134] Ryan J.L., Heckler C.E., Ling M., Katz A., Williams J.P., Pentland A.P., Morrow G.R. (2013). Curcumin for Radiation Dermatitis: A Randomized, Double-Blind, Placebo-Controlled Clinical Trial of Thirty Breast Cancer Patients. Radiat. Res..

[135] Patel P.B., Thakkar V.R., Patel J.S. (2015). Cellular Effect of Curcumin and Citral Combination on Breast Cancer Cells: Induction of Apoptosis and Cell Cycle Arrest. J. Breast Cancer.

[136] Nencioni A., Caffa I., Cortellino S., Longo V.D. (2018). Fasting and cancer: Molecular mechanisms and clinical application. Nat. Rev. Cancer.

[137] Lee C., Raffaghello L., Brandhorst S., Safdie F.M., Bianchi G., Martin-Montalvo A., Pistoia V., Wei M., Hwang S., Merlino A. (2012). Fasting Cycles Retard Growth of Tumors and Sensitize a Range of Cancer Cell Types to Chemotherapy. Sci. Transl. Med..

[138] Senderowicz A.M. (1999). Flavopiridol: The First Cyclin-Dependent Kinase Inhibitor in Human Clinical Trials. Investig. New Drugs.

[139] Asghar U., Witkiewicz A.K., Turner N.C., Knudsen E.S. (2015). The history and future of targeting cyclin-dependent kinases in cancer therapy. Nat. Rev. Drug Discov..

[140] Tan A.R., Swain S.M. (2002). Review of flavopiridol, a cyclin-dependent kinase inhibitor, as breast cancer therapy. Semin. Oncol..

[141] Murphy C.G., Dickler M.N. (2015). The Role of CDK4/6 Inhibition in Breast Cancer. Oncologist.

[142] Ghia P., Scarfò L., Perez S., Pathiraja K., DeRosier M., Small K., McCrary Sisk C., Patton N. (2017). Efficacy and safety of dinaciclib vs ofatumumab in patients with relapsed/refractory chronic lymphocytic leukemia. Blood.

[143] Parry D., Guzi T., Shanahan F., Davis N., Prabhavalkar D., Wiswell D., Seghezzi W., Paruch K., Dwyer M.P., Doll R. (2010). Dinaciclib (SCH 727965), a Novel and Potent Cyclin-Dependent Kinase Inhibitor. Mol. Cancer Ther..

[144] Hu S., Marineau J.J., Rajagopal N., Hamman K.B., Choi Y.J., Schmidt D.R., Ke N., Johannessen L., Bradley M.J., Orlando D.A. (2019). Discovery and Characterization of SY-1365, a Selective, Covalent Inhibitor of CDK7. Cancer Res..

[145] Patel H., Periyasamy M., Sava G.P., Bondke A., Slafer B.W., Kroll S.H.B., Barbazanges M., Starkey R., Ottaviani S., Harrod A. (2018). ICEC0942, an Orally Bioavailable Selective Inhibitor of CDK7 for Cancer Treatment. Mol. Cancer Ther..

[146] Johannessen L.H., Hu S., Ke N., D'Ippolito A., Rajagopal N., Marineau J., Savinainen A., Zamboni W., Hodgson G. (2019). Abstract C091: Preclinical evaluation of PK, PD, and antitumor activity of the oral, non-covalent, potent and highly selective CDK7 inhibitor, SY-5609, provides rationale for clinical development in multiple solid tumor indications. Mol. Cancer Ther..

[147] Zhang Z., Peng H., Wang X., Yin X., Ma P., Jing Y., Cai M.-C., Liu J., Zhang M., Zhang S. (2017). Preclinical Efficacy and Molecular Mechanism of Targeting CDK7-Dependent Transcriptional Addiction in Ovarian Cancer. Mol. Cancer Ther..

[148] Abella A., Dubus P., Malumbres M., Rane S.G., Kiyokawa H., Sicard A., Vignon F., Langin D., Barbacid M., Fajas L. (2005). Cdk4 promotes adipogenesis through PPARγ activation. Cell Metab..

[149] Walker A.J., Wedam S., Amiri-Kordestani L., Bloomquist E., Tang S., Sridhara R., Chen W., Palmby T.R., Zirkelbach J.F., Fu W. (2016). FDA Approval of Palbociclib in Combination with Fulvestrant for the Treatment of Hormone Receptor–Positive, HER2-Negative Metastatic Breast Cancer. Clin. Cancer Res..

[150] Hamilton E., Cortes J., Dieras V., Ozyilkan O., Chen S.-C., Petrakova K., Manikhas A., Jerusalem G., Hegg R., Lu Y. (2019). Abstract PD1-11: NextMONARCH 1: Phase 2 study of abemaciclib plus tamoxifen or abemaciclib alone in HR+, HER2- advanced breast cancer. Cancer Res..

[151] Shah A., Bloomquist E., Tang S., Fu W., Bi Y., Liu Q., Yu J., Zhao P., Palmby T.R., Goldberg K.B. (2018). FDA Approval: Ribociclib for the Treatment of Postmenopausal Women with Hormone Receptor–Positive, HER2-Negative Advanced or Metastatic Breast Cancer. Clin. Cancer Res..

[152] Finn R.S., Dering J., Conklin D., Kalous O., Cohen D.J., Desai A.J., Ginther C., Atefi M., Chen I., Fowst C. (2009). PD 0332991, a selective cyclin D kinase 4/6 inhibitor, preferentially inhibits proliferation of luminal estrogen receptor-positive human breast cancer cell lines in vitro. Breast Cancer Res..

[153] Finn R.S., Cristofanilli M., Ettl J., Gelmon K.A., Colleoni M., Giorgetti C., Gauthier E., Liu Y., Lu D.R., Zhang Z. (2020). Treatment effect of palbociclib plus endocrine therapy by prognostic and intrinsic subtype and biomarker analysis in patients with bone-only disease: A joint analysis of PALOMA-2 and PALOMA-3 clinical trials. Breast Cancer Res. Treat..

[154] Wardell S.E., Ellis M.J., Alley H.M., Eisele K., VanArsdale T., Dann S.G., Arndt K.T., Primeau T., Griffin E., Shao J. (2015). Efficacy of SERD/SERM Hybrid-CDK4/6 Inhibitor Combinations in Models of Endocrine Therapy–Resistant Breast Cancer. Clin. Cancer Res..

[155] Malorni L., Curigliano G., Minisini A.M., Cinieri S., Tondini C., Arpino G., Pavesi L., Martignetti A., Criscitiello C., Puglisi F. (2017). A phase II trial of the CDK4/6 inhibitor palbociclib (P) as single agent or in combination with the same endocrine therapy (ET) received prior to disease progression, in patients (pts) with hormone receptor positive (HR+) HER2 negative (HER2−) metastatic breast cancer (mBC) (TREnd trial). J. Clin. Oncol..

[156] Masuda N., Inoue K., Nakamura R., Rai Y., Mukai H., Ohno S., Hara F., Mori Y., Hashigaki S., Muramatsu Y. (2019). Palbociclib in combination with fulvestrant in patients with hormone receptor-positive, human epidermal growth factor receptor 2-negative advanced breast cancer: PALOMA-3 subgroup analysis of Japanese patients. Int. J. Clin. Oncol..

[157] Konecny G.E., Hendrickson A.E.W., Jatoi A., Burton J.K., Paroly J., Glaspy J.A., Dowdy S.C., Slamon D.J. (2016). A multicenter open-label phase II study of the efficacy and safety of palbociclib a cyclin-dependent kinases 4 and 6 inhibitor in patients with recurrent ovarian cancer. J. Clin. Oncol..

[158] DeMichele A., Clark A.S., Tan K.S., Heitjan D.F., Gramlich K., Gallagher M., Lal P., Feldman M., Zhang P., Colameco C. (2015). CDK 4/6 Inhibitor Palbociclib (PD0332991) in Rb^+^Advanced Breast Cancer: Phase II Activity, Safety, and Predictive Biomarker Assessment. Clin. Cancer Res..

[159] Konecny G.E., Winterhoff B., Kolarova T., Qi J., Manivong K., Dering J., Yang G., Chalukya M., Wang H.-J., Anderson L. (2011). Expression of p16 and Retinoblastoma Determines Response to CDK4/6 Inhibition in Ovarian Cancer. Clin. Cancer Res..

[160] Vaughn D.J., Hwang W.T., Lal P., Rosen M.A., Gallagher M., O’Dwyer P.J. (2015). Phase 2 trial of the cyclin-dependent kinase 4/6 inhibitor palbociclib in patients with retinoblastoma protein-expressing germ cell tumors. Cancer.

[161] Liu T., Yu J., Deng M., Yin Y., Zhang H., Luo K., Qin B., Li Y., Wu C., Ren T. (2017). CDK4/6-dependent activation of DUB3 regulates cancer metastasis through SNAIL1. Nat. Commun..

[162] Gelbert L.M., Cai S., Lin X., Sanchez-Martinez C., Del Prado M., Lallena M.J., Torres R., Ajamie R.T., Wishart G.N., Flack R.S. (2014). Preclinical characterization of the CDK4/6 inhibitor LY2835219: In-Vivo cell cycle-dependent/independent anti-tumor activities alone/in combination with gemcitabine. Investig. New Drugs.

[163] Tate S.C., Sykes A.K., Kulanthaivel P., Chan E.M., Turner P.K., Cronier D.M. (2018). A Population Pharmacokinetic and Pharmacodynamic Analysis of Abemaciclib in a Phase I Clinical Trial in Cancer Patients. Clin. Pharmacokinet..

[164] Patnaik A., Rosen L.S., Tolaney S.M., Tolcher A.W., Goldman J.W., Gandhi L., Papadopoulos K.P., Beeram M., Rasco D.W., Hilton J.F. (2016). Efficacy and Safety of Abemaciclib, an Inhibitor of CDK4 and CDK6, for Patients with Breast Cancer, Non-Small Cell Lung Cancer, and Other Solid Tumors. Cancer Discov..

[165] Dickler M.N., Tolaney S.M., Rugo H.S., Cortés J., Diéras V., Patt D., Wildiers H., Hudis C.A., O'Shaughnessy J., Zamora E. (2017). MONARCH 1, A Phase II Study of Abemaciclib, a CDK4 and CDK6 Inhibitor, as a Single Agent, in Patients with Refractory HR+/HER2− Metastatic Breast Cancer. Clin. Cancer Res..

[166] Kim E.S. (2017). Abemaciclib: First Global Approval. Drugs.

[167] McAndrew N.P., Dickson M.A., Clark A.S., Troxel A.B., O’Hara M.H., Colameco C., Gallager M., Gramlich K., Zafman K., Vaughn D. (2020). Early treatment-related neutropenia predicts response to palbociclib. Br. J. Cancer.

[168] Iwata H., Umeyama Y., Liu Y., Zhang Z., Schnell P., Mori Y., Fletcher O., Marshall J.-C., Johnson J.G., Wood L.S. (2021). Evaluation of the Association of Polymorphisms With Palbociclib-Induced Neutropenia: Pharmacogenetic Analysis of PALOMA-2/-3. Oncologist.

[169] Gelmon K., Walshe J.M., Mahtani R., Joy A.A., Karuturi M., Neven P., Lu D.R., Kim S., Schnell P., Bananis E. (2021). Efficacy and safety of palbociclib in patients with estrogen receptor–positive/human epidermal growth factor receptor 2–negative advanced breast cancer with preexisting conditions: A post hoc analysis of PALOMA-2. Breast.

[170] Johnston S., Puhalla S., Wheatley D., Ring A., Barry P., Holcombe C., Boileau J.F., Provencher L., Robidoux A., Rimawi M. (2019). Randomized Phase II Study Evaluating Palbociclib in Addition to Letrozole as Neoadjuvant Therapy in Estrogen Receptor–Positive Early Breast Cancer: PALLET Trial. J. Clin. Oncol..

[171] Hurvitz S.A., Martin M., Press M.F., Chan D., Fernandez-Abad M., Petru E., Rostorfer R., Guarneri V., Huang C.-S., Barriga S. (2020). Potent Cell-Cycle Inhibition and Upregulation of Immune Response with Abemaciclib and Anastrozole in neoMONARCH, Phase II Neoadjuvant Study in HR^+^/HER2^−^ Breast Cancer. Clin. Cancer Res..

[172] Sledge G.W., Toi M., Neven P., Sohn J., Inoue K., Pivot X., Burdaeva O., Okera M., Masuda N., Kaufman P.A. (2020). The Effect of Abemaciclib Plus Fulvestrant on Overall Survival in Hormone Receptor–Positive, ERBB2-Negative Breast Cancer That Progressed on Endocrine Therapy—MONARCH 2: A Randomized Clinical Trial. JAMA Oncol..

[173] Sledge G.W., Toi M., Neven P., Sohn J., Inoue K., Pivot X., Burdaeva O., Okera M., Masuda N., Kaufman P.A. (2017). MONARCH 2: Abemaciclib in Combination With Fulvestrant in Women With HR+/HER2− Advanced Breast Cancer Who Had Progressed While Receiving Endocrine Therapy. J. Clin. Oncol..

[174] Johnston S.R.D., Harbeck N., Hegg R., Toi M., Martin M., Shao Z.M., Zhang Q.Y., Rodriguez J.L.M., Campone M., Hamilton E. (2020). Abemaciclib Combined With Endocrine Therapy for the Adjuvant Treatment of HR+, HER2−, Node-Positive, High-Risk, Early Breast Cancer (monarchE). J. Clin. Oncol..

[175] (2020). Abemaciclib Reigns Over Breast Cancer in MonarchE. Cancer Discov..

[176] Tolaney S.M., Beeram M., Beck J.T., Conlin A., Dees E.C., Puhalla S.L., Rexer B.N., Burris H.A., Jhaveri K., Helsten T. (2022). Abemaciclib in Combination With Endocrine Therapy for Patients With Hormone Receptor-Positive, HER2-Negative Metastatic Breast Cancer: A Phase 1b Study. Front. Oncol..

[177] Dey N., Williams C., Williams K., Klein J., Carlson J.H., Starks D., Espaillat L.R., De P., Jones B.L. (2018). Abstract 3451: Testing signaling algorithm in platinum-resistant ovarian carcinomas: A simultaneous inhibition of RAS-RAF and cell-cycle pathways signals by trametinib with paclitaxel or ribociclib/abemaciclib. Cancer Res..

[178] Peguero J.A., O'Neil B.H., Sohal D., Bauer T.M., Subbiah V., Kelly K., Grilley-Olson J.E., Nadauld L., Safran H., Slosberg E.D. (2016). Genomic mutation profiling (GMP) and clinical outcome in patients (pts) treated with ribociclib (CDK4/6 inhibitor) in the Signature program. J. Clin. Oncol..

[179] Colon-Otero G., Zanfagnin V., Hou X., Foster N.R., Asmus E.J., Hendrickson A.W., Jatoi A., Block M.S., Langstraat C.L., Glaser G.E. (2020). Phase II trial of ribociclib and letrozole in patients with relapsed oestrogen receptor-positive ovarian or endometrial cancers. ESMO Open.

[180] Colon-Otero G., Weroha S.J., Zanfagnin V., Foster N.R., Asmus E., Hendrickson A.E.W., Jatoi A., Block M.S., Langstraat C.L., Glaser G.E. (2019). Results of a phase 2 trial of ribociclib and letrozole in patients with either relapsed estrogen receptor (ER)-positive ovarian cancers or relapsed ER-positive endometrial cancers. J. Clin. Oncol..

[181] Slamon D.J., Neven P., Chia S., Fasching P.A., De Laurentiis M., Im S.-A., Petrakova K., Bianchi G.V., Esteva F., Martín M. (2020). Overall Survival with Ribociclib plus Fulvestrant in Advanced Breast Cancer. N. Engl. J. Med..

[182] Slamon D.J., Neven P., Chia S., Fasching P.A., De Laurentiis M., Im S.-A., Petrakova K., Bianchi G.V., Esteva F.J., Martín M. (2018). Phase III randomized study of ribociclib and fulvestrant in hormone receptor-positive, human epidermal growth factor receptor 2-negative advanced breast cancer: MONALEESA-3. J. Clin. Oncol..

[183] Tripathy D., Sohn J., Im S.-A., Colleoni M., Franke F., Bardia A., Harbeck N., Hurvitz S., Chow L., Lee K. (2018). Abstract GS2-05: First-line ribociclib vs placebo with goserelin and tamoxifen or a non-steroidal aromatase inhibitor in premenopausal women with hormone receptor-positive, HER2-negative advanced breast cancer: Results from the randomized phase III MONALE. Cancer Res..

[184] Slamon D.J., Fasching P.A., Patel R., Verma S., Hurvitz S.A., Chia S.K.L., Crown J., Martin M., Barrios C.H., Spera G. (2019). NATALEE: Phase III study of ribociclib (RIBO) + endocrine therapy (ET) as adjuvant treatment in hormone receptor–positive (HR+), human epidermal growth factor receptor 2–negative (HER2–) early breast cancer (EBC). J. Clin. Oncol..

[185] Tan A.R., Wright G.S., Thummala A.R., Danso M.A., Popovic L., Pluard T.J., Han H.S., Vojnović Z., Vasev N., Ma L. (2019). Trilaciclib plus chemotherapy versus chemotherapy alone in patients with metastatic triple-negative breast cancer: A multicentre, randomised, open-label, phase 2 trial. Lancet Oncol..

[186] Iyengar M., O’Hayer P., Cole A., Sebastian T., Yang K., Coffman L., Buckanovich R.J. (2018). CDK4/6 inhibition as maintenance and combination therapy for high grade serous ovarian cancer. Oncotarget.

[187] Curigliano G., Pardo P.G., Meric-Bernstam F., Conte P., Lolkema M., Beck J., Bardia A., Garcia M.M., Penault-Llorca F., Dhuria S. (2016). Ribociclib plus letrozole in early breast cancer: A presurgical, window-of-opportunity study. Breast.

[188] Malorni L., Piazza S., Ciani Y., Guarducci C., Bonechi M., Biagioni C., Hart C.D., Verardo R., Di Leo A., Migliaccio I. (2016). A gene expression signature of retinoblastoma loss-of-function is a predictive biomarker of resistance to palbociclib in breast cancer cell lines and is prognostic in patients with ER positive early breast cancer. Oncotarget.

[189] Gong X., Litchfield L.M., Webster Y., Chio L.-C., Wong S.S., Stewart T.R., Dowless M., Dempsey J., Zeng Y., Torres R. (2017). Genomic Aberrations that Activate D-type Cyclins Are Associated with Enhanced Sensitivity to the CDK4 and CDK6 Inhibitor Abemaciclib. Cancer Cell.

[190] Finn R.S., Crown J.P., Lang I., Boer K., Bondarenko I.M., Kulyk S.O., Ettl J., Patel R., Pinter T., Schmidt M. (2015). The cyclin-dependent kinase 4/6 inhibitor palbociclib in combination with letrozole versus letrozole alone as first-line treatment of oestrogen receptor-positive, HER2-negative, advanced breast cancer (PALOMA-1/TRIO-18): A randomised phase 2 study. Lancet Oncol..

[191] Bartlett C.H., Mardekian J., Cotter M.J., Huang X., Zhang Z., Parrinello C.M., Bourla A.B. (2020). Concordance of real-world versus conventional progression-free survival from a phase 3 trial of endocrine therapy as first-line treatment for metastatic breast cancer. PLoS ONE.

[192] Yu Q., Geng Y., Sicinski P. (2001). Specific protection against breast cancers by cyclin D1 ablation. Nature.

[193] Teo Z.L., Versaci S., Dushyanthen S., Caramia F., Savas P., Mintoff C.P., Zethoven M., Virassamy B., Luen S.J., McArthur G. (2017). Combined CDK4/6 and PI3Kα Inhibition Is Synergistic and Immunogenic in Triple-Negative Breast Cancer. Cancer Res..

[194] Asghar U., Barr A., Cutts R., Beaney M., Babina I., Sampath D., Giltnane J., Lacap J.A., Crocker L., Young A. (2017). Single-Cell Dynamics Determines Response to CDK4/6 Inhibition in Triple-Negative Breast Cancer. Clin. Cancer Res..

[195] Martin M., Zielinski C., Ruiz-Borrego M., Carrasco E., Turner N., Ciruelos E.M., Muñoz M., Bermejo B., Margeli M., Anton A. (2020). Palbociclib in combination with endocrine therapy versus capecitabine in hormonal receptor-positive, human epidermal growth factor 2-negative, aromatase inhibitor-resistant metastatic breast cancer: A phase III randomised controlled trial—PEARL. Ann. Oncol..

[196] Liao X., Hong Y., Mao Y., Chen N., Wang Q., Wang Z., Zhang L., Wang L., Shi C., Shi W. (2020). SPH3643: A novel cyclin-dependent kinase 4/6 inhibitor with good anticancer efficacy and strong blood-brain barrier permeability. Cancer Sci..

[197] Lu J.F., Zhu M.Q., Xie B.C., Shi X.C., Liu H., Zhang R.X., Xia B., Wu J.W. (2022). Camptothecin effectively treats obesity in mice through GDF15 induction. PLOS Biol..

